# Comparative transcriptomic analysis of male and females in the dioecious weeds *Amaranthus palmeri* and *Amaranthus tuberculatus*

**DOI:** 10.1186/s12870-023-04286-9

**Published:** 2023-06-26

**Authors:** Lucas K. Bobadilla, Yousoon Baek, Patrick J. Tranel

**Affiliations:** Department of Crop Sciences, University of Illinois, Urbana, IL USA

**Keywords:** Dioecy, Sex-determination, Reproduction, Weeds, RNA-seq, Sex-determinant region, Waterhemp, Palmer amaranth

## Abstract

**Background:**

Waterhemp (*Amaranthus tuberculatus* (Moq.) Sauer) and Palmer amaranth (*Amaranthus palmeri* S. Wats.) are two dioecious and important weed species in the world that can rapidly evolve herbicide-resistance traits. Understanding these two species' dioecious and sex-determination mechanisms could open opportunities for new tools to control them. This study aims to identify the differential expression patterns between males and females in *A. tuberculatus* and *A. palmeri*. Multiple analyses, including differential expression, co-expression, and promoter analyses, used RNA-seq data from multiple tissue types to identify putative essential genes for sex determination in both dioecious species.

**Results:**

Genes were identified as potential key players for sex determination in *A. palmeri.* Genes *PPR247*, *WEX,* and *ACD6* were differentially expressed between the sexes and located at scaffold 20 within or near the male-specific Y (MSY) region. Multiple genes involved with flower development were co-expressed with these three genes. For *A. tuberculatus,* no differentially expressed gene was identified within the MSY region; however, multiple autosomal class B and C genes were identified as differentially expressed and possible candidate genes.

**Conclusions:**

This is the first study comparing the global expression profile between males and females in dioecious weedy *Amaranthus* species. Results narrow down putative essential genes for sex-determination in *A. palmeri* and *A. tuberculatus* and also strengthen the hypothesis of two different evolutionary events for dioecy within the genus.

**Supplementary Information:**

The online version contains supplementary material available at 10.1186/s12870-023-04286-9.

## Background

Contemporary agriculture must evolve to meet the challenges of achieving high yields sustainably to feed a growing population. Among these challenges, weed management continues to be of high priority due to the constant evolution and adaptation of weeds to chemical management practices [1, 2]. Collectively, waterhemp (*Amaranthus tuberculatus* (Moq.) Sauer) and Palmer amaranth (*Amaranthus palmeri* S. Wats.) are considered pervasive weed species throughout much of the US. Recent surveys by the Weed Science Society of America (WSSA) classified these two weeds as the most troublesome in corn and two of the three most troublesome in soybean [3].

Waterhemp and Palmer amaranth are herbaceous, annual plants native to the American Midwest and Southwest, respectively [4], and with many attributes contributing to weediness, including high photosynthetic rates, rapid growth rates, and germination periods that extend throughout much of the growing season [5, 6]. However, the main attribute that makes these weeds so troublesome is their ability to evolve resistance rapidly and repeatedly to herbicides [7]: to date, waterhemp and *A. palmeri* have evolved resistance to herbicides spanning seven and eight sites of action, respectively [8].

The *A. tuberculatus* and *A. palmeri* weediness characteristics are shared by many of the other *Amaranthus* weeds [6, 9]. However, setting *A. tuberculatus* and *A. palmeri* apart is their dioecy characteristic. Whereas most other *Amaranthus* species are monoecious and predominantly self-pollinated, separate female and male plants of the dioecious species ensure outcrossing. This high outcrossing rate promotes genetic diversity, rapid adaptation, and efficient "stacking" of multiple herbicide-resistance traits, leaving producers with few effective herbicide choices [10, 11].

Dioecy can be defined as the presence of male and female individuals within a plant species. Dioecy is not common, present in about 6% of angiosperms [12, 13]. Despite its infrequency, however, dioecy has evolved independently in several lineages and is found in many important species [e.g., spinach (*Spinacia oleracea*), asparagus (*Asparagus* spp.), hops (*Humulus* spp.), cannabis (*Cannabis* spp.), kiwifruit (*Actinidia* spp.), papaya (*Carica* *papaya*), fig (*Ficus* *carica*), persimmon (*Diospyros* *kaki*), grape (*Vitis* *vinifera*), strawberry (*Fragaria* spp.) and willow (*Salix* spp.)] [12, 14–17]. The dioecy condition is derived from ancestral hermaphrodite flowers, and the evolution of dioecy has multiple and independent pathways across a diversity of angiosperm species [14, 18–20]. Two possible venues have been hypothesized for the evolution of dioecious plants from hermaphrodite ancestors: via monoecious or gynodioecious (i.e., female and hermaphrodite plants co-exist within the species) pathways [21].

Dioecy provides several advantages and potential disadvantages to the success of a species. Separate sexes enforce outcrossing, favor high heterozygosity and associated heterosis, and can lead to plants being more efficient in their reproductive role [21]. For example, female plants could invest more energy into seed production, providing more resources to their progeny [22]. An obvious disadvantage of being dioecious is that a single plant cannot colonize a new area. Also, as population density decreases, the chances of reproductive success decrease, which could lead to local extinction. This disadvantage can potentially be used to control dioecious weeds by removing one sex within an area via a gene drive approach, leading to the local extinction of populations [23, 24]. However, for this gene-drive weed management approach to be developed, the first step is to identify the sex determination mechanism within the species.

Identification of sex-determination genes in dioecious plants is inherently challenging. The lack of recombination within the sex-specific region precludes genetic mapping, sex-specific genes are derived from — and therefore homologous to — genes that might also be present in the other sex, and accumulation of highly repetitive DNA sequences due to non-recombination hinders the analysis of the sex-specific region [25]. Despite these challenges, progress is being made for several species.

For instance, *Silene* *latifolia* (white campion) is a model for studying sex chromosome evolution. It has heteromorphic sex chromosomes and a male heterogametic system (XY) [26, 27]. Several genes have been discussed as potential sex-determining factors: *S. latifolia* X/Y-gene 1 (*SIX/Y1*), encoding a WD-repeat protein and likely involved in cell proliferation, *SlX/Y4*, encoding a fructose-2,6-bisphosphatase [28], the floral organ identity gene *APETALA3* (*SlAP3*) [29], which is specifically involved in the development of androecia, and orthologs of *SHOOT MERISTEMLESS* (*STM*) (named *SlSTM1* and *SlSTM2*) and *CUP-SHAPED COTYLEDON 1* (*CUC1*) and *CUC2* [30–32], which are both activators of cytokinin biosynthesis [33]. A physical map of the Y chromosome has narrowed down the regions carrying the sex-determination genes [34], and identifying the pseudo-autosomal boundaries [35] should greatly aid in gene identification. Recently, a gene specific to males in white campion named *CLAVATA-3* was also identified as a major gynoecium repressor [36].

Papaya (*Carica papaya*) has also emerged as a model for dioecy [14]. This species is sub-dioecious in that males, females, and hermaphrodites exist. Sub-dioecy, in this case, is due to two different Y regions: one resulting in males (XY) and the other in hermaphrodites (XY^h^) [37]. Kiwifruit (*A. deliciosa*) is a major fruit crop with an XY system of sex determination. Studies in kiwifruit demonstrated a two-factor model for sex determination, with one gene affecting ovule production and another pollen production. A male-specific type-C cytokinin response regulator called *SHY GIRL* (*SyGI*) was identified as a suppressor of feminization. More recently, a second Y-encoded gene named *Friendly Boy* (*FrBy*) was found to cause male flower production. Those two genes were found to act independently as female suppressor and male promoter genes, respectively [38–40].

A similar system as found in kiwifruit was documented in asparagus (*A. officinalis*), which contains two sexually antagonistic genes in linkage disequilibrium on the Y chromosome. The first gene is the Y-specific *SUPPRESSOR OF FEMALE FUNCTION* (*SOFF*), which acts as a suppressor of femaleness, while the second gene, *aspTDF1*, acts as a male promotion agent [15].

Although the two-factor model is well accepted, it is not the only path to dioecy; sex determination can also occur via a single-factor model in which the factors regulating female and male function are connected by an epistatic genetic interaction rather than physical linkage [41, 42]. This single factor model for sex determination is considered a monomorphic pathway in which the dioecy system potentially evolves from hermaphroditism through a population including monoecious individuals [22, 43]. Interestingly, one of the most characterized dioecious species uses a single-factor model. Both single- and two-factor models can occur within a genus. For instance, the *Populus* genus has at least two distinct sex-determining regions located in the same chromosome among multiple *Populus* species, suggesting multiple dioecy evolutionary events within a genus [44–46].

Regarding sex determination in dioecious *Amaranthus* spp., several decades ago it was reported that sex determination is under genetic control, with males and females typically occurring in a 1:1 ratio and males being the heterogametic sex [47]. Cytological observations have failed to identify heteromorphic sex chromosomes in dioecious amaranths [48], suggesting a relatively small region of a chromosome determines sex. Because all known *Amaranthus* species are monoecious or dioecious, dioecy is thought to have evolved from monoecy and is thought to be relatively recent since dioecious and monoecious species can produce viable hybrids [4, 49]. Recent advances were made to identify male-specific Y (MSY) regions within *A. tuberculatus* and *A. palmeri* utilizing male-specific markers and the draft genomes. The MSY regions in *A. palmeri* and *A. tuberculatus* span about 1.3 Mbp and 4.6 Mbp, respectively. Consistent with the hypothesis that dioecy evolved separately in the two species, synteny was not detected between the two species' MSY regions [50–52]. Even though the MSY identification led to a considerable advance towards elucidating the dioecious mechanism, no precise candidate gene was identified. This gap can be filled by transcriptomics comparisons between sexes to narrow down further putative candidates for sex-determination in dioecious weedy *Amaranthus* species. In this paper, we report a comprehensive transcriptomics comparison between females and males in both *A. tuberculatus* and *A. palmeri* utilizing transcriptome assemblies from floral and vegetative tissues to identify putative essential genes for sex determination.

## Results

### Transcriptome assembly and reads quantification

Twenty-four 150 bp pair-read libraries were sequenced for each species using Illumina Novaseq 6000 technology. From each sex of each species, three tissue types, each with four biological replicates, were collected for RNA extraction. Tissues were shoot-apical-meristem (SAM), floral meristem, and mature flowers. After data preprocessing, an average of 63 million reads were obtained per library (Supplemental table 1). In order to conduct the transcriptome comparison in both species, a genome-guided de novo transcriptome assembly was conducted using Trinity for each species [53] (Table 1). Assembled transcripts were further annotated using Uniprot and Pfam databases, and the top three hits from each database were returned. Reads were pseudo-aligned back to transcriptomes using Kallisto [54]. The final transcript-expression count matrix was summarized to gene level prior to analysis, leading to a final read-count matrix containing 40,857 genes for *A. tuberculatus* and 26,121 genes for *A. palmeri* that were used for further analyses.

**Table 1:** Transcriptome assembly statistics

					**Trinity**		**Functionally annotated**	
**Species**	**Average Contig (bp)**	**N50 (bp)**	**BUSCO score (%)**	**GC (%)**	**transcripts**	**genes**	**transcripts**	**genes**
*A. tuberculatus*	1193.28	2,293	99.7	36.78	782,414	321,945	234,086	40,857
*A. palmeri*	1596.21	3,055	97.7	36.37	441,665	176,412	203,372	26,121

### *Amaranthus palmeri* transcriptome comparison

#### Principal component analysis

A principal component analysis (PCA) was conducted to identify the overall expression patterns and driver genes. Quantified read counts were loaded into R, summarized to gene level, and normalized via DESeq2 method for analysis [55]. PC plots were generated by mapping samples based on sex and tissue to identify major driver genes.

Scree plot showed that PCs 1 to 3 explained 80% of expression variation within *A. palmeri* samples. A clear separation across tissues was noticed between SAM, floral meristem, and mature male flowers. However, female flowers were not well differentiated from the floral meristem tissues, indicating a greater variation in expression in male flower transition. PC3 and PC2 reduced the distance between mature flowers, clustering all samples closely (Supplemental figure 1).

The top 30 gene loadings for the first 3 PCs were extracted (Supplemental figure 2). Among top-loading in PC1 and PC2 were the genes *Scarecrow-like 32*, *ACCELERATED CELL DEATH 6 (ACD6)*, *HOTHEAD*, *MEN-8*, and EP2/ERF transcription factors MADS-box *SOC1* and *AGL24*, which are known to modulate class B and C homeotic genes regulating flowering time and floral tissue identity [56, 57]. Other enriched genes, such as the pollen tube guide *FERONIA*, were previously associated with pollen development [58]. For PC3, multiple genes involved with chromatin remodeling, transposons, and post-transcriptional regulation were identified, indicating the potential involvement of these mechanisms in sex identity for *A. palmeri* [59, 60].

#### Differential expression analysis

To identify the differentially expressed (DE) genes, a differential expression analysis was conducted within the previously generated gene-level read-count matrix from both species using EdgeR [61]. Contrasts were generated to compare between sexes for each tissue and between tissues within each sex (Table 2). Genes with low expression in more than 80% of samples were filtered, and the remaining genes were subjected to normalization via the trimmed mean of M-values (TMM) method. Over-represented and conditional gene ontology (GO)-term enrichment analyses were conducted using TopGO to identify enriched terms [62].

**Table 2:** Pairwise comparisons made in all analyses for both *Amaranthus* species

Across sex			Within sex		
**Tissue 1**		**Tissue 2**	**Tissue 1**		**Tissue 2**
♂ Mature flower	vs	♀ Mature flower	♂ SAM	vs	♂ Floral meristem
♂ Floral meristem	vs	♀ Floral meristem	♂ SAM	vs	♂ Mature flower
♂ SAM	vs	♀ SAM	♂ Floral meristem	vs	♂ Mature flower
			♀ SAM	vs	♀ Floral meristem
			♀ SAM	vs	♀ Mature flower
			♀ Floral meristem	vs	♀ Mature flower

**Fig. 1:**
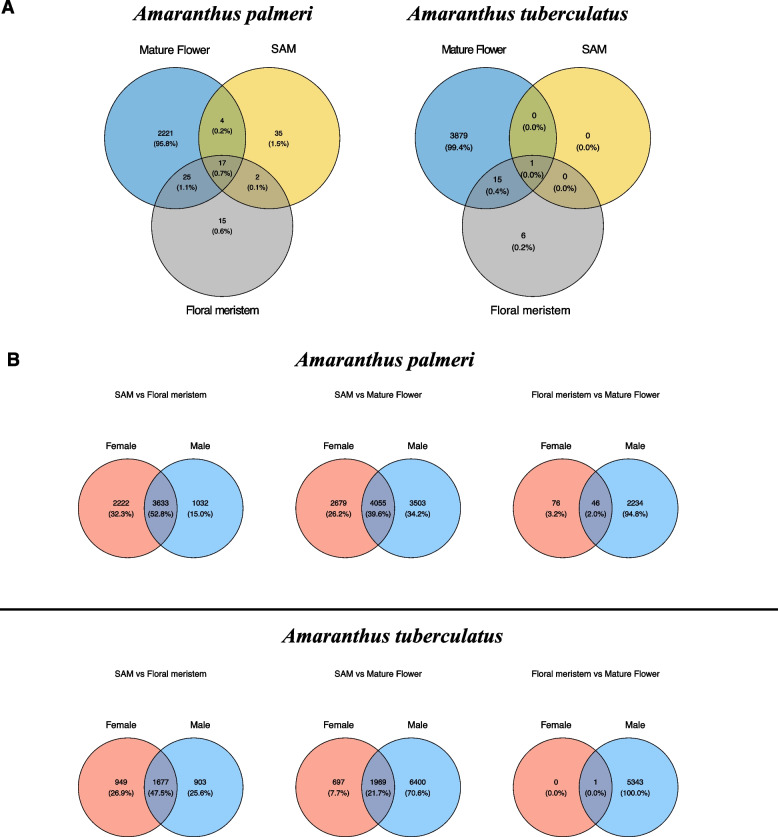
Venn diagrams showing numbers of differentially expressed genes (% of total). **A** differentially expressed genes between sexes for each tissue type. **B** differentially expressed genes between tissue types within each sex. SAM; shoot apical meristem

**Fig. 2:**
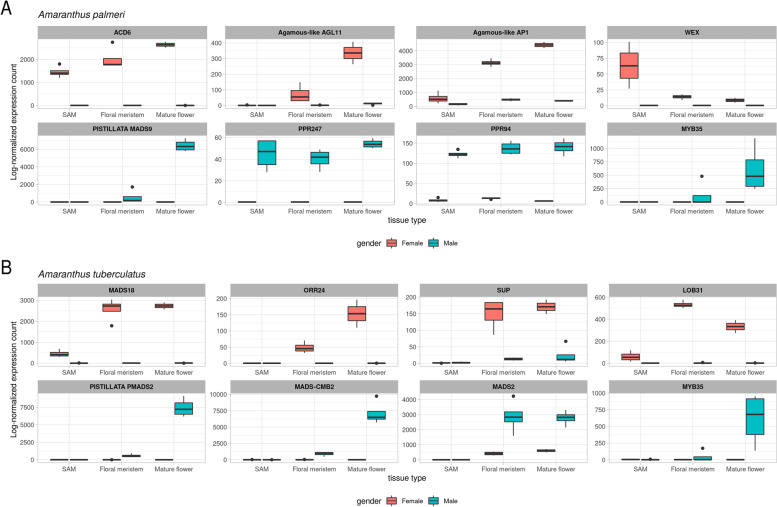
Log-normalized expression profile in *Amaranthus palmeri* (**A**) of genes for the two pentatricopeptide repeat-containing proteins (PPR), *PPR247* and *PPR94*, the accelerated cell death protein (*ACD6*), Werner Syndrome-like exonuclease (*WEX*), Agamous-like *AP1*, PISTILLATA (*MADS9*) and *MYB35*. **B** Log-normalized expression profile of *Amaranthus tuberculatus* genes for lateral-organ boundary 31 (*LOB31*), MADS-box protein *CMB2*, MADS-box transcription factor 18 (*MADS18*), floral homeotic protein *PISTILLATA* (*PMADS2*), *SUPERMAN* (*SUP*) and two-component response regulator 24 (*ORR24*), *MYB35* and Agamous MADS-box (*MADS2*)

As observed in the PCA, the comparison across tissues showed the most significant number of DE genes, indicating a considerable influence of tissue type over the expression profile. The comparisons between sexes showed that the major differential expression is observed in mature flowers, while less than 100 genes were DE between the sexes in the two other tissue types. Among the DE genes between the sexes, about 1% (17) were DE in all three tissue types (Fig. 1). Two genes encoding pentatricopeptide repeat-containing proteins (PPR), *PPR247* and *PPR94*, were highly up-regulated in all male tissues (Fig. 2), and *ACD6* and *Werner Syndrome-like exonuclease* (*WEX*) were highly up-regulated in females (Fig. 2).

When mapped to the *A. palmeri* genome, *PPR247*, *ACD6*, and *WEX* aligned to scaffold 20, where the MSY region was predicted [51]. *PPR247* was located within the MSY region, *ACD6* was located at the beginning of the scaffold, and *WEX* was identified at 200 kb before the predicted MSY starting point. *PPR94* was mapped to one of the male-specific regions [51] in scaffold 259.

Within floral meristems, 59 genes were DE between the sexes (38 up-regulated and 21 down-regulated in males). The most significant was *ACD6*, which was down-regulated in all male tissues. The *PPR247* and *PPR94* genes were also highly up-regulated in males in this comparison (> 8 and > 3 logFC, respectively). Other key genes annotated with flower development function were also identified, such as AGAMOUS MADS-box *AP1*, lateral organ boundary (*LOB31*), and *PISTILLATA MADS2-like*. *AP1* was also located within scaffold 20, where the MSY region is located, while the other genes were located within autosomal regions.

Enrichment analysis was conducted to identify key genes differentiating female and male mature flowers (Supplemental table 2). A total of 219 GO terms were significant in the enrichment analyses, with 33 GO terms (comprised of 431 genes) involving flower development, floral identity, or hormone responses. Within the up-regulated genes in males compared to females, *PPR94* and *PPR247* were identified as well as multiple genes involved in tapetum and pollen maturation, such as the transcriptional activator PHD-finger *MALE STERILITY 1* [63, 64], the transcription factor *GAMYB* [65], tapetum and pollen formation regulator *MYB80* [66], *FLOWERING LOCUS C*, and multiple transcription factors from the bHLH family [67, 68]. Down-regulated genes in males compared to females included the two-component response regulator 24 (*ORR24*) involved in cytokinin regulation, *LOB31*, gibberellin-regulated 4 (*GASA4*), *ACD6*, *WEX*, and the transcription activator *SHI RELATED SEQUENCE 1* (*SHI*), which is known to be involved in multiple flowering processes such as regulation of gynoecium, stamen and flowering timing [69]. Multiple DE genes were enriched for the term "maintenance of meristem identity," such as *SOC1*, *CLAVATA2*, *AP2*, and *AGL24* [57, 70–72]. Interestingly, two genes encoding *SOC1* were identified as being DE, one up-regulated (located in scaffold 13) and the other down-regulated (located in scaffold 80) in male flowers, indicating potential sex-regulated *SOC1* copies in late floral development.

The comparisons between tissue types within sex highlight interesting patterns (Fig. 1). Within each sex, when comparing SAM with the other two tissue types, all comparisons yielded many DE genes (> 5,000 genes). The male SAM and mature flower comparison yielded the highest number of DE genes, comprising about 20% of the transcriptome, indicating a significant shift in expression pattern during male floral development. Enrichment analyses were conducted to identify reproduction and hormone-related terms within each sex.

SAM and floral meristem comparisons were used to identify early flowering process genes uniquely DE for males or females. Within this comparison, 2222 DE genes were DE only in the female comparison. These genes were identified in 37 scaffolds, with 878 within scaffold 1, including genes involved with general organogenesis, hormone signaling, and stress responses (Supplemental table 1). Interestingly, scaffolds 8 and 4 each contained a set of physically clustered genes involved with meristem identity, flower development, and hormone signaling. A total of 195 genes were associated with the shoot system development term, including *LOB31*, *FT-interacting 1*, *AP1*, and *ORR24*. Within the male SAM/floral meristem comparison, a total of 195 terms were enriched, including terms involved with pollen development, stamen and petal identity specification, and hormone-related terms. Some key genes identified were *ACD6*, *SHI*, floral homeotic *PMADS 2*, *BEL-1*, and the transcriptional corepressor *LEUNIG*.

For the SAM and mature flower comparison, a total of 2,679 genes were uniquely identified within the female comparison, from which enrichment analysis identified 225 significant GO terms associated with flowering processes (Supplemental table 2). For the comparison within the male sex, 3,503 DE genes with 216 enriched terms were identified, most involving pollen development and auxin-related terms. Interestedly, terms involved with epigenetic modifications, such as miRNA silencing, histone modifications, and RNA-directed DNA methylation, were identified, highlighting their potential importance for flower development [73, 74].

Floral meristem and mature flower comparison within sexes were used to identify late-flowering genes unique for males or females (Fig. 1). Interestingly, of the 2356 genes DE between floral meristems and mature flowers, only 76 (3%) were unique to females. Genes uniquely DE in the female comparison included a class A floral gene *AP2/ERF AINTEGUMENTA* (*ANT*), located at Scaffold 6 and involved with the fusion of carpels and of medial ridges leading to ovule primordia [75–77]. From the genes uniquely DE in the male comparison, enrichment analysis indicated 233 significant terms with some involved with floral development or hormone responses. Three hundred twenty-two genes were associated with those key terms, including multiple genes encoding *MYB* transcription factors, auxin- and gibberellin-related proteins, and multiple pollen-development-related proteins. Genes encoding proteins involved with epigenetic regulation were also identified within this comparison.

#### Co-expression network analysis

A weighted co-expression network analysis (WGCNA) was conducted using the WGCNA package [78] to identify gene co-expression patterns and correlate them with sex and tissues. Read counts were normalized using the DESeq2 method and converted to a log2 scale prior to fitting to WGCNA models. Genes with high co-expression patterns were further assigned to expression groups denominated as modules named using colors. From each module, hub genes (genes having high connectivity with most other genes within the module) were identified via intra-modular analysis correlating gene-trait significance and module membership. Module-trait associations were made by running a biweight midcorrelation within module eigengenes and the comparisons previously described (Table 2).

**Fig. 3:**
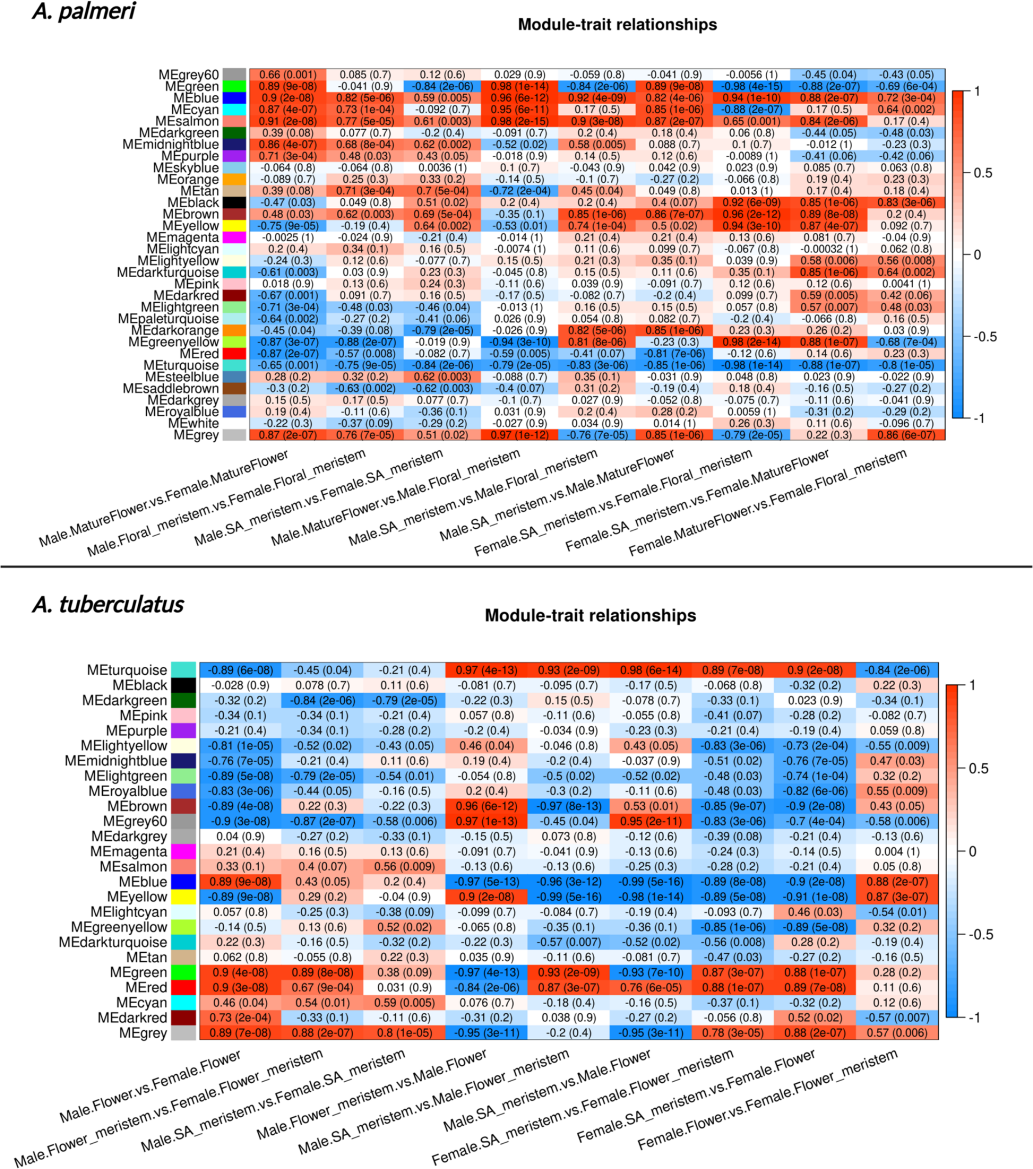
Weighted co-expression network analysis module-trait correlation from two dioecious *Amaranthus* species. Values represent correlation values within each comparison, with *p*-values for the biweight midcorrelation test shown in parenthesis. Each column refers to a comparison described in Table 1, with red colors indicating a correlation bias to males and blue a bias to females for the between-sex comparisons (columns 1 to 3). For tissue comparison within sexes (columns 4 to 9), red colors indicate a correlation towards the first tissue and blue colors a correlation towards the second tissue listed in the comparison

A total of 32 modules were identified, ranging from 57 to 3051 genes within each module (Supplemental figure 3 and Supplemental table 3). Module eigengene values correlate each module with all comparisons where for each comparison, the first level of the comparison name was used as the reference level (Fig. 3). Modules Blue, Cyan, and Salmon had a clear correlation (biweight midcorrelation; *p*-value ≤ 0.05) with almost all comparisons. Two other modules, Tan and Brown, were highly correlated with both SAM and floral meristem comparisons between sexes. The Brown module was positively correlated with most comparisons indicating that it potentially carries genes more related to tissue differentiation. Interestingly, modules Red and Turquoise were highly negatively correlated with most comparisons, and since male tissues were used as the reference groups in all between-sex comparisons, these are likely female-biased modules. The correlation within modules was also estimated via the Pearson correlation model (Supplemental figure 4).

The Tan module showed a clear male-biased pattern (Fig. 3 and Supplemental figure 5), due to the constant large eigenvalues in male tissues and included some key genes identified in the differential expression analysis, such as *PPR247* and *PPR94*. Another interesting candidate within the Tan module is the gene *MYST* which encodes a histone acetyltransferase protein and was already characterized as an epigenetic regulator for flowering in other species by the repression of the genes *FLOWERING LOCUS C* and other MADS-box genes [79, 80]. Similar to *MYST*, two other genes encoding proteins involved in flower epigenetic regulation were identified within the Tan module: genes encoding the two histone methyltransferases ASHR1 and ASHR2 (Supplemental figure 6), which are known to play a crucial role in reproduction organ development and chromatin remodeling [81].

**Fig. 4:**
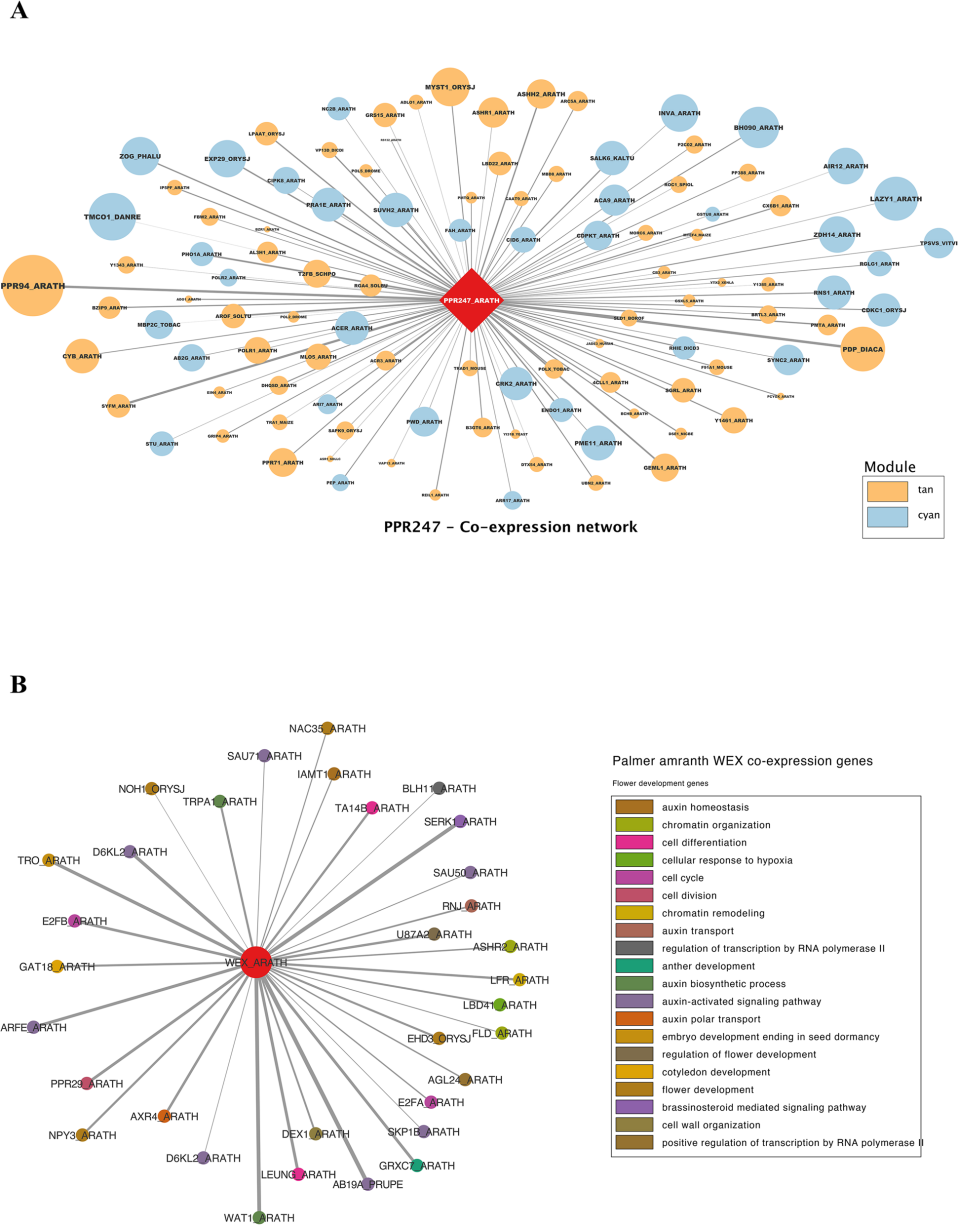
Co-expression networks obtained for key genes hypothesized to play sex-determination roles in *Amaranthus palmeri*. **A** *PPR247* co-expression network across genes from two correlated modules*.* **B** *Werner Syndrome-like exonuclease* (*WEX*) gene co-expression network with genes enriched for flowering-related terms. Edge size indicates the co-expression weights

Among the genes within the Tan module, 50 were found to be DE in at least one comparison. The primary genes identified within this final list were *PPR247*, *PPR94*, and *LOB22*. The genes *MYST*, *ASHR2*, and *PPR247,* were identified as hubs. Since *PPR247* was also identified as male-specific, a network was built to identify genes co-expressed with it using genes from the Tan module and the Cyan module due to its correlation with the Tan module (Fig. 4). Within genes highly correlated with *PPR247*, *PPR94* was highly co-expressed with *MYST*, *ASHR2*, *ASHR1*, and *LOB22*.

The Blue module was highly associated with all tissue comparisons; however, since it encompassed 3,051 genes, a GO term enrichment analysis was conducted to identify the primary GO terms. A total of 273 GO terms were significant, from which genes containing terms involved with floral development, hormones, programmed cell death, and transcription regulation were further analyzed. Within those terms, 305 genes were identified and included genes encoding multiple MADS-box proteins, ERF transcription factors, flowering-promoting factors, and LOB, BEL1, and NAC transcription factors**.** Genes identified as hubs included the transcription factors *NAC56*, *NAC73*, *ERF34*, a *LOB*, *MADS4*, *MYB36*, and *MYB58* (Supplemental figure 7). Most genes identified as keys for the Blue module network topology were flowering-related. Intra-module analysis showed that the Blue module carries essential genes for flower development in both sexes, with a clear bias toward males. The central hub within the Blue module is the class B gene encoding a MADS4 protein, whose homologs were already characterized in other species to play critical roles in flower identity [82]. *MADS4* is located within scaffold 81, close to a region containing male-specific reads [51]. Among Blue module genes, 1718 were DE in at least one comparison and 1131 genes had enriched GO terms. A LOB-encoding gene within scaffold 7 was identified as a hub and was only DE for floral meristem and mature flower comparisons to SAM in males. *MADS4*, *ERF34*, *MYB*s, and *BEL1* were DE in both female and male comparisons indicating that they play roles for flowering development in both sexes.

For the Red module, most enriched terms were found to be associated with photosynthesis and chloroplast development and had only three genes associated with the term negative regulation of flower development. Interestingly, the *ACD6* gene identified previously in the DE analysis, involved in programmed cell death, and located close to the MSY region in scaffold 20, was part of this female-biased module.

The Turquoise module was identified as a major module related to floral development, from which enrichment analysis identified 482 genes related to flowering, hormonal responses, and plant organ development. One of the key genes identified was *APETALA AP2*, a transcriptional activator that promotes early floral meristem identity and regulation of *AGAMOUS* genes and recruiting the *TOPLESS* gene, which was also present within the Turquoise module [83]. One of the major hubs in the Turquoise module was annotated as homologous to the rice gene *CRL5*, which was found to work in the repression of cytokinin signaling by positively regulating the two-component response regulator [84]. *CRL5* was co-expressed with another hub in this module, *ORR24*. The floral-development genes *SOC1* and MADS-Box *AGL24* were also identified as hubs, with both having roles in flower identity [57, 70].

**Fig. 5:**
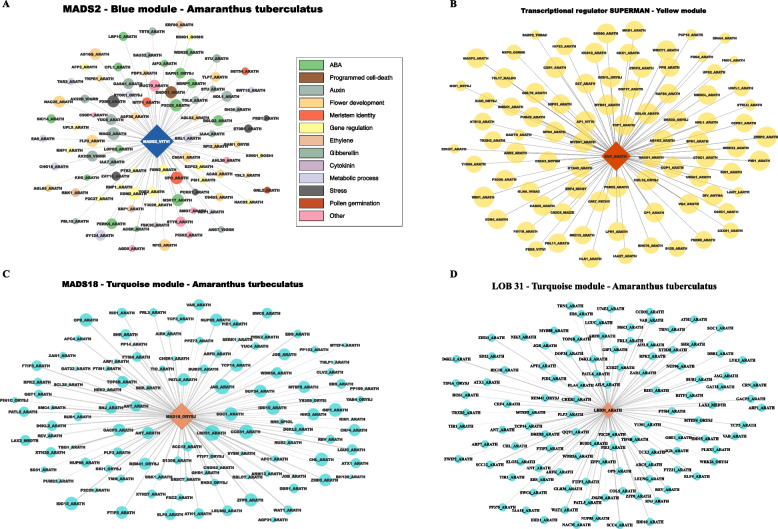
*Amaranthus tuberculatus* co-expression networks for the hub genes **A** *PISTILLATA MADS2*, **B** *SUPERMAN*, **C** *MADS18*, and D: *LOB31*. Node colors in (**A**) refer to the related function of each gene, while in **B**, **C** and **D** refer to the module from which the gene was assigned. The size of the nodes represents their gene-trait significance, and edges’ transparency indicates gene connectivity

The *WEX* gene was found within the Turquoise module, which contains essential genes for floral development and tissue identity, indicating its potential function for female flower identity. From genes highly co-expressed (weight > 0.15) with *WEX*, multiple were functionally annotated as post-transcriptional epigenetic regulators or involved with chromatin modeling, floral identity, flowering time, or gametophyte formation (Fig. 5).

Another interesting set of genes within the Turquoise module include the *HEN1*, *HEN2*, and *HEN4* genes, which are known to work in association with floral determinacy specification via *AGAMOUS* regulation, mRNA degradation, and sRNA methylation, repressing class B and C genes [85–87]. *HEN1* encodes an enhancer of genes *HUA1* and *HUA2*, which specify reproductive organ identities, repress A gene function, and can control floral determinacy and carpel formation via sRNA silencing [88–91]. *HEN2* encodes a DExH-box RNA helicase that acts redundantly with *HEN1*, *HUA1*, and *HUA2* in the specification of floral organ identity in the third whorl; *HEN4* encodes a K homology (KH) domain-containing, putative RNA-binding protein that interacts with *HUA1*, a CCCH zinc finger RNA-binding protein in the nucleus [85]. Multiple copies of *HEN1*and *HEN4* were identified, and a single *HEN2*. Regarding expression patterns, none of the *HEN* genes were differentially expressed in any of the between-sex comparisons, indicating that they are equally important in both sexes. On the other hand, one of the copies each of *HEN4* and *HEN1* were only DE in the female SAM vs. mature flower comparison.

#### Trans factor and promoter analysis

Promoter analysis **(**Supplemental table 4) was conducted using the list of identified DE and essential genes to identify enriched motifs for predicting regulatory function and putative transcription factors (TF) involved. Promoter sequences (1500 bp before the TSS) were used in PlantRegMap [92] *Arabidopsis thaliana* transcription factors motif database. The regulatory prediction was made via an over-representation of transcription factor motif enrichment.

A total of 182 TFs were found enriched for DE genes. Results point to multiple MYB TF motifs being enriched within the DE genes in the floral meristem tissue comparison between sexes. The most significant hits were motifs from the TF *MYB30*, *MYB31*, and *MYB22*, all known to be involved with tissue differentiation and flower development. Other TF with enriched motifs **(**Supplemental figure 8**)** included a member from the TEP family (bHLH binding protein–protein interaction) known to play a role in flower symmetry, and the HD-ZIP family, which can function in meristem identity, organ development, and hormonal responses.

One hundred fifty TF motifs were enriched within the DE genes’ promoter regions for the SAM comparison between sexes. Most enriched TF were from the MYB family, followed by TCP and MADS-box families. This gene was annotated as *TSO1*, which is required for male and female tissue development [93]. *TSO1* was found to have an identical expression pattern in both male and female tissues, being up-regulated in floral meristem tissues, indicating that this gene works as a general TF in both sexes for flower development. However, the top enriched motif TF was from the CPP family, which is known to play an essential role in developing reproductive tissue and controlling cell division in plants.

MADS-box and MYB TF motifs were also largely predicted among the promoter regions. Even though no DE MYB TF was detected in the SAM comparison, *MYB 35* and *MYB36* were identified as highly up-regulated in mature male flowers, indicating a potential role in maintaining the expression of crucial genes later in flower development.

A total of 13,935 potential regulatory interactions between 599 TFs and 469 DE genes were identified for DE genes in mature flower tissue comparison between sexes, with 279 TFs having over-represented motifs in their promoter regions. Among the top five enriched TFs for DE genes in mature flower comparison, three MYBs, one MADS-box, and one GATA were found. The most significant TF, with 86 binding motifs, was annotated as *MYB30*, which is known to play a role in programmed cell death, flower development, and flowering time control. The genes *MYB35* and *MYB36* were among the most up-regulated genes in mature male flowers. Among the top TFs, motifs for the MADS-box genes AGAMOUS *AGL15*, *AGL66*, and *MADS2* were all among the DE genes.

Among the critical genes identified within all analyses, the promoter regions from those genes were analyzed for predicting the putative regulatory agents. *PPR247* was identified with motifs for multiple ERF TFs indicating a likely regulation by ERF TF. Interestingly, a specific MADS-box motif was identified 25 base pairs before the *PPR247* TSS. This motif was predicted to be the binding site for the MADS-box genes *SEPALLATA/PISTILLATA* and *AP1*, indicating a likely regulation by those genes. Both *AP1* and *PISTILLATA* were identified among the DE genes.

### *Amaranthus tuberculatus* transcriptome comparison

#### Principal component analysis

Like *A. palmeri*, *A. tuberculatus* had significant expression variation primarily attributed to mature male flowers and represented by PC1. PC2 represented the differences across the other tissues (Supplemental figure 9). PC3, on the other hand, mainly described experimental variation across replications. Among the significant loading genes (Supplemental figure 4) for the first three PCs, the majority were related to pollen development. Genes involved in programmed cell death were identified for PC1 loading. Another gene among PC1 loading genes and in common with *A. palmeri* was the gene *AP2-like* ethylene-responsive transcription factor, indicating its importance for flower identity [94]. Within PC2 loading genes, more genes in common with *A. palmeri* PCA were identified, including the MADS-box genes *SOC1* and *AGL24*. Multiple other genes encoding MADS-box proteins, including *AP1* and *AGL31*, previously characterized as important genes for flower development [70, 95], were identified in *A. tuberculatus*.

#### Differential expression analysis

Similar to the results observed from the *A. palmeri* differential expression analysis, the largest number of DE genes between sexes was found in mature flowers (Fig. 1 and Supplemental tab). Also similar to *A. palmeri*, SAM and floral meristem tissue comparisons between sexes of *A. tuberculatus* yielded a low number of differentially expressed genes. In fact, only one gene was DE when comparing SAM tissues between sexes and was down-regulated in males. This gene was annotated as *MADS-box 18* (*MADS18*), which was previously characterized as involved in multiple flowering processes, including floral identity [96].

From the floral meristem tissue comparison between sexes, 23 DE genes were identified, including *MADS18* (Fig. 2). The genes *PISTILLATA/MADS2* (*MADS2*), *AGAMOUS*, *SOC1*, a bHLH transcription factor, MYB-like transcription factor *EOBII*, and *LOB* 31 were also DE and play critical roles in floral development [57, 67, 97]. Most genes were down-regulated in males except for *MADS2*, *CYP710A1*, MADS-box *CMB2*, and transcription factors *bHLH60* and *bHLH91*. All the 23 genes were also DE in mature flowers. The gene encoding the floral homeotic protein MADS2 was predominantly up-regulated in male floral tissues and is homologous to *Petunia hybrida MADS2* and the *PISTILLATA MADS9* (*MADS9*) from *Vitis vinifera,* which was previously characterized as a class B transcription factor that might play a role in specifying stamen and petal organ identity [98, 99].

Among the enriched terms for the mature flower results, the most general term found was reproduction and was accompanied by flowering development, hormone-related, transcriptional regulation, and post-transcriptional regulation (Supplemental table 5). On hundred thirty two DE genes were enriched with the flower development GO term. The DE genes leucine-rich repeat receptor-like serine/threonine-protein kinase (*BAM1*), cullin-1 (*CUL1*), *SEUSS*, *LEUNIG*, agamous-like MADS-box protein *AGL15*, *SHI*, ethylene-responsive transcription factor (*CRF2*), transcription factor (*DUO1*), and *MADS18* were among the DE genes with enriched GO terms. Most of the enriched DE genes were down-regulated in males and are mainly involved in gynoecium and female embryo development based on annotations.

Comparisons between tissues of each sex were conducted to identify DE genes at early and late tissue transition that are unique for each sex (Fig. 1). From SAM and floral meristem comparisons, 949 genes were uniquely DE in the female comparison, while 903 genes were unique to males. Within the set of unique female genes, 114 GO terms were enriched, including the key terms cytokinin-activated signaling pathway, response to auxin, regulation of transcription, and regulation of flower development. These 114 enriched GO terms were comprised of 235 genes, including *ARR24*, *LOB31*, *MADS18*, multiple ERF transcription factors, and the class A transcriptional regulator *STERILE APETALA* (*SAP*). Another interesting gene was the transcriptional regulator *SUPERMAN* (*SUP*), which was found to be up-regulated in female floral meristem and mature flowers (Fig. 2) and known to prevent the B class homeotic proteins from acting in the gynoecia whorl and also up-regulated in females in multiple dioecious species [100–103].

For the male-unique set of DE genes, about 280 terms were enriched, including multiple pollen development terms, specification of floral organ identity, and positive regulation of flower development. Among genes annotated with flowering-related terms were *ABORTED MICROSPORES, SOC1, AP2, ANT, BEL1*, and the gene *HEN2*, involved in the maintenance of homeotic B and C gene expression in the reproductive whorls [104]. DE genes were mapped to the *A. tuberculatus* genome; however, no specific DE genes with clear floral identity roles were identified within the contigs previously identified as a putative part of the *A. tuberculatus* MSY region [51].

Comparison between floral meristem and mature flower mainly yielded genes uniquely DE in males. This was expected due to PCA results showing that mature flowers were overlapping with floral meristems in females while mature male flowers were leading the main PC, indicating that male flower development could occur earlier than in females, leading to more prominent differential expression in mature male flowers.

#### Co-expression network analysis

The WGCNA results for *A. tuberculatus* analysis yielded 24 modules ranging from 4340 to 49 genes per module (Supplemental figure 3 and Supplemental table 6). Unlike the *A. palmeri* analysis, no module showed a clear correlation pattern across all comparisons; however, some patterns can be observed (Fig. 3). For instance, female-biased modules such as Lightgreen and Grey60 were identified among all tissue comparisons. Other modules were female-biased within only a specific tissue comparison across the sexes, such as Lightyellow, Yellow, and Darkgreen modules. Some male-biased modules were also identified, especially in the mature flower comparison across sexes, such as the Green, Red, Cyan, and Darkred modules.

The Turquoise module was correlated with most tissue comparisons, indicating genes essential for flowering development in both sexes (Fig. 3). The Turquoise module contained floral identity-related genes identified in the DE analysis, such as PISTILLATA-like *SOC1, MADS18,* and *LOB31*. Blue and Yellow modules also appeared to be late-flowering developing modules and contained critical genes such as the DE AGAMOUS MADS-box, *MADS2*, and *SUP* genes.

The Blue module was one of the most significant modules, the one to which most DE genes were assigned, and was identified as a male-bias module. The Blue module was enriched for multiple flowering-related terms. A total of 517 DE genes were located within the Blue module, with 93 genes enriched for some flowering-related GO term (Supplemental figure 13). Genes included *agamous-like MADS2* and the floral homeotic *AGAMOUS*, both of which were also identified as hub genes. Another identified hub gene was *UFO*, which is known to positively regulate class B genes and negatively regulate class C genes [105]. *UFO* was also previously found to be a downstream gene for sex determination in poplar, in which an ARR gene represses it, triggering female organ development [106]. Among the critical genes within the Blue module, the *AGAMOUS-like MADS2* was among the hub genes. Based on connectivity, the most co-expressed genes with *AGAMOUS-like MADS2* were the transcription factor *NAC92*, highly involved with programmed cell-death and flowering senescence, the *NRT1/ PTR FAMILY 5.2, UFO*, and multiple flowering/hormone-related genes (Fig. 5).

The Brown module also contained a large number of genes that were DE. Analysis showed that this module was mainly enriched for terms related to ABA, regulation of transcription, and anther development terms. The brown module contained 105 DE genes, including SHORT INTERNODES (*SHI*), *CLAVATA*, and *WUSHEL*, which are known to play a role in late gynoecium and anther development [71]. *SHI* was identified as one of the main hubs within the Brown module, indicating that the Brown module likely contains genes co-expressed along the flower development process in both sexes.

The Turquoise module was significantly correlated with multiple comparisons (Fig. 3), indicating that essential flowering genes were assigned to it. It contained 215 DE genes, including the female-specific *MADS18*. Enrichment analysis from the Turquoise module identified multiple terms related to flower development, regulation of meristem development, and megagametogenesis. Interestingly, the genes *MADS18* and *LBD31* were identified as two outliers among the hub genes with high gene-trait significance correlation in the intra-modular analysis (> 0.99 correlation); however, the genes were found with low module membership indicating that those genes are likely specific for their function and removing them would not affect the overall network topology. Among the hub genes, the transcription factor *TCP5* was identified, which plays a pivotal role in the control of morphogenesis of shoot organs and ovule development by negatively regulating the expression of boundary-specific genes via miRNA regulation [107]. Other transcription factors were identified as hubs: the corepressor *SMAX1-LIKE 3* and the Zinc finger protein *JAGGED* control the morphogenesis of lateral organs and flower whorl shape [108, 109]. For the two central genes within this module, *MADS18*, and *LOB31*, a network with the most connected genes was built to identify the central genes co-expressed with them (Fig. 5). Both networks included genes involved with gynoecium, including *SOC1*, *JAGGED*, and *HEN2*, which regulate classes B and C via miRNA, and the *ANT* gene, which is crucial for developing gynoecium marginal tissues [85]. The Turquoise module captured multiple genes involved with gynoecium and flower organ patterning, indicating potential candidates for a female inducer gene.

#### Trans factor and promoter analysis

The same promoter analysis conducted for *A. palmeri* was conducted for *A. tuberculatus* (Supplemental table 4). For the floral meristem tissue between-sex comparison, 917 potential regulatory relationships between 338 TFs and 25 DE genes were identified. From those identified TFs, 167 possess over-represented motif targets in the DE genes. The top ten enriched motifs were eight MADS-box TFs and two MYB TFs. The most significant TF was an *AGL16* MADS-box gene, which participates in the repression of FT expression and floral transition. The second most significant TF was a floral homeotic protein AGAMOUS, which is involved in the control of organ identity during the early development of flowers [101]. The two MYB TFs identified were annotated as *MYB96*, which encodes an *R2R3* type MYB TF whose expression is strongly induced by abscisic acid, and MYB61, which coordinates a small network of downstream target genes required for several aspects of plant growth and development [110]. Other AGAMOUS MADS-box TFs were also identified, including *SEPALLATA*, *PISTILLATA*, and *AGL13*, which play a crucial role in gametophyte morphogenesis [111].

A total of 4,989 potential regulatory relationships between 580 TFs and 265 genes were identified from the DE genes identified in the mature flower comparison between sexes, from which 265 TFs possess over-represented target motifs (Supplemental table 6). MYB was the most present family (Supplemental figure 16). The top three consisted of the TFs *MYB15* and *MYB13*, known to be involved in enhancing expression levels of genes involved with abscisic acid signaling, and a TF from the SRS family SHI, which was found up-regulated in mature female flowers. A TF from the AP2 family was also identified among the most enriched. The TF family ERF was the second most frequent among the enriched TF predicted regulation. ERF has essential functions in the transcriptional regulation of various biological processes related to growth and development and various responses to environmental stimuli [112].

**Fig. 6:**
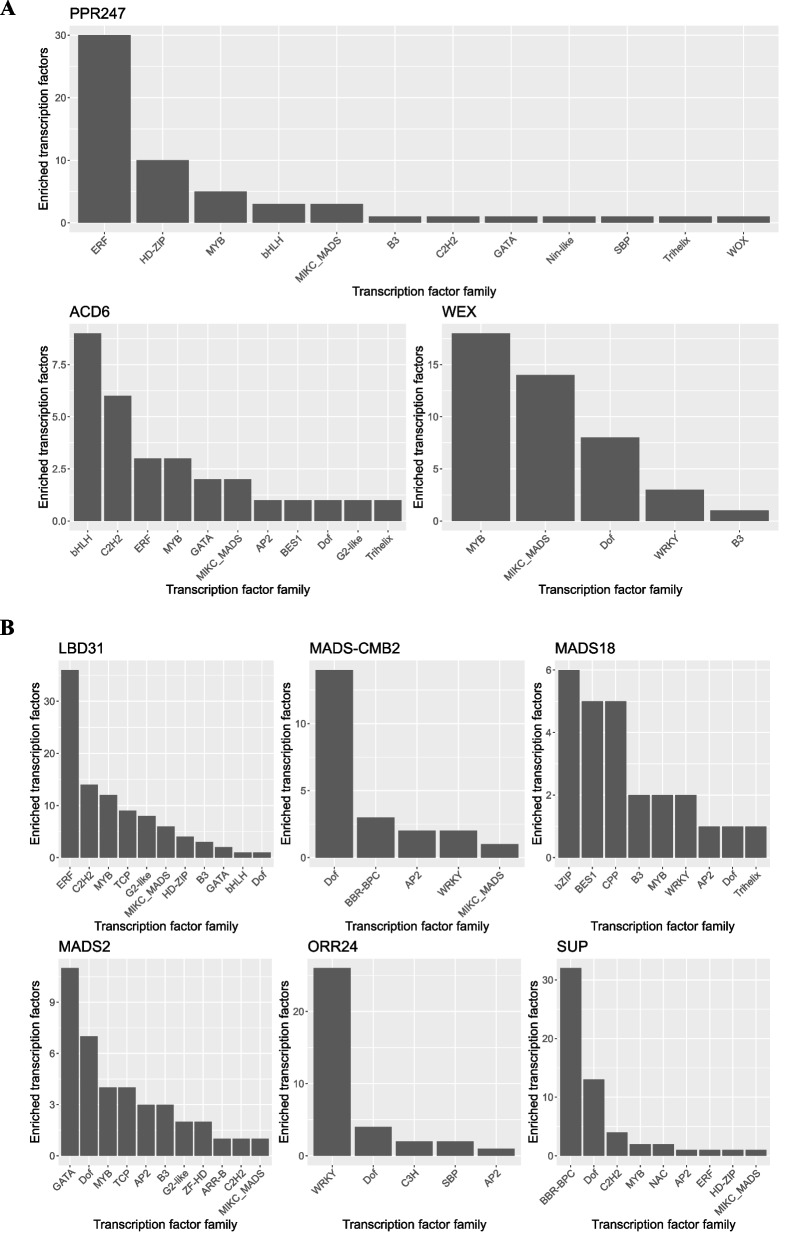
Motif enrichment analysis results for the *PPR247*, *ACD6*, and *WEX* genes in *Amaranthus palmeri* (**A**) and the *LBD31*, *MADS-CMB2*, *MADS18*, and *MADS2*, *ORR24*, and *SUP* genes in *Amaranthus tuberculatus* (**B**). The y-axis displays the total number of transcription factors per family that had motifs enriched in each gene promoter region

Promoter analysis was done on each of six main genes identified in *A. tuberculatus* analysis (Fig. 6). A total of 148 specific TF motifs in the promoter region were identified. Interestingly, all genes showed different predicted regulatory patterns. For instance, *MADS18* showed many motifs for bZIP TF while *ORR24* showed mainly WRKY TF motifs. *PMADS2*, one of the most up-regulated *PISTILLATA* in male tissues, also showed the presence of MADS-box motifs, indicating a potential auto-regulation. *ORR24* was identified with some distinct motifs, such as SBP TF, which is involved in development and floral organogenesis. Other interesting patterns were noticed, such as the significant presence in the *SUP* promoter region of motifs for the BBR-BPC TF family, which is known to be involved in developmental processes [113].

#### MADS-box and LOB evolutionary analysis

**Fig. 7:**
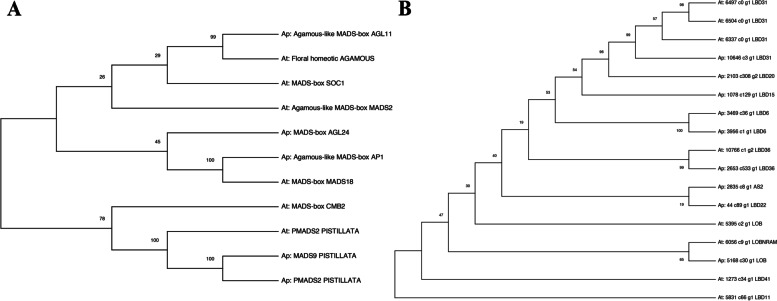
The evolutionary history of differentially expressed MADS-box genes (**A**) and LOB genes (**B**) in *Amaranthus palmeri* (Ap) and *Amaranthus tuberculatus* (At). Protein sequences were used to fit the Maximum Likelihood method and Le Gascuel model. Values represent the percentage of bootstraps (*n* = 500) from the fitted model, indicating the proximity level across protein sequences

Multiple MADS-boxes and LOB genes were DE and co-expressed with multiple flowering development genes in both species, indicating a potential commonality. Phylogeny trees were built using the protein sequences from all DE genes encoding MAD-box and LOB of *A. palmeri*, and A. *tuberculatus* (Fig. 7). Analysis was conducted in MEGA11 [114] using the Maximum Likelihood method and Le Gascuel model [115]. The bootstrap consensus tree inferred from 500 replicates [116] represents the evolutionary proximity between the sequences indicating putative similarities.

Results from MADS-box phylogeny show interesting similarities across the two species. For instance, the most differentially expressed MADS-box in *A. tuberculatus*, annotated as homologous to MADS-box 18 (*MADS18*), was identified with strong similarities with the *A. palmeri* AGAMOUS-like AP1 (*AP1*) located at scaffold 20. *MADS18* and *AP1* were up-regulated in female mature flowers and floral meristematic tissues, suggesting that both MADS-box genes play a similar role in gynoecium development in both species. Also, there was 45% bootstrap support for phylogeny between these two MADS-box genes and another MADS-box gene from *A. palmeri* homologous to *AGL24*, an essential transcription activator that mediates floral transition and floral tissue identity working in a dosage-dependent way, mainly when associated with an *AP1* gene [117–119].

Two other MADS-box genes identified with high evolutionary similarities (99% bootstrap support) were the *A. palmeri AGL11* and the *A. tuberculatus* floral homeotic *AGAMOUS* gene. These two genes were also up-regulated in female floral tissues. Previous studies with *AGL11* in *Vitis vinifera* identified it as the major gene for seedless cultivars, indicating that this gene may play a role later in the development of embryos and seeds in both *Amaranthus* species [120]. Another *A. tuberculatus* MADS-box identified with some divergence from *AGL11* and *AGAMOUS* (29% bootstrap) was annotated as homologous to *SOC1*; however, this gene was differentially expressed only in the within-sex tissue comparisons.

A set of MADS box genes annotated as *PISTILLATA* were also conserved across the two species. Two *PISTILLATA* genes from *A. palmeri, MADS2*, and *MADS9*, were highly similar, indicating a potential duplication event since both were mapped to scaffold 80 but with different coordinates. Also, both genes were up-regulated in male tissues, and *MADS2* was among one of the most up-regulated genes in *A. palmeri* between-sex comparisons. The *A. tuberculatus MADS2 PISTILLATA* gene was highly similar to *A. palmeri MADS2* and *MADS9* (100% bootstrap support) and was also highly up-regulated in male compared to female floral tissues. Previous studies characterized *PISTILLATA* genes as central class B genes, indicating their importance for staminate flower formation and their potential role at the end of the male flower formation pathway [98, 121, 122].

For LOB phylogeny topology, additional ramifications were noticed. A more significant number of LOB genes were differentially expressed in *A. palmeri* compared to *A. tuberculatus* (Fig. 7). Results show that most LOBs from *A. palmeri* have multiple copies in the genome. For instance, three copies of *LOB31* were identified in *A. palmeri, one* showing considerable divergence from the other two (57% bootstrap), with all three predominantly up-regulated in female floral tissues. Intriguingly, the *A. tuberculatus LOB31* was highly similar to the three *LOB31* genes from *A. palmeri* and showed the same expression pattern with up-regulation in female floral tissues. *LOB36* showed strong similarities between the two species and was up-regulated in male floral tissues in both species. 

## Discussion

The dioecious nature of *A. palmeri* and *A. tuberculatus* provides them a robust evolutionary advantage as weeds making them very adaptable to agricultural management practices [123]. Understanding the nature of the dioecy mechanism of these two species could ultimately yield strategies to manipulate sex as a novel genetic control strategy. Our results indicate a putative single-gene model within the previously identified MSY for sex determination in *A. palmeri,* while for *A. tuberculatus,* results indicate the involvement of crucial autosomal TFs.

### *Amaranthus palmeri* single-gene mechanism model

Results for *A. palmeri* show distinct differentiation regarding the expression patterns between females and males. Transcriptome comparative results point to a sex determination mechanism involving a male fertility restorer gene (*PPR247*) in males and a post-transcriptional regulation gene (*WEX*) with a controlled cell death mechanism (*ACD6*) in females. *PPR247* was only expressed in male tissues (Fig. 2) and was identified in a previously characterized male-specific region [51]. PPR is an interesting candidate due to its previous characterization as a major cytoplasmatic male fertility restorer [124]. PPR genes are also necessary for embryogenesis, indicating another key reproduction role [125]. The fact that it is exclusively expressed in males and present in the MSY region could indicate that it can serve as an inducer for male organ formation by blocking genes repressing male organ formation. Co-expression network analysis showed that *PPR247* is co-expressed with multiple flower-related genes (Fig. 4), indicating its likely involvement with male fertility restoration.

**Fig. 8:**
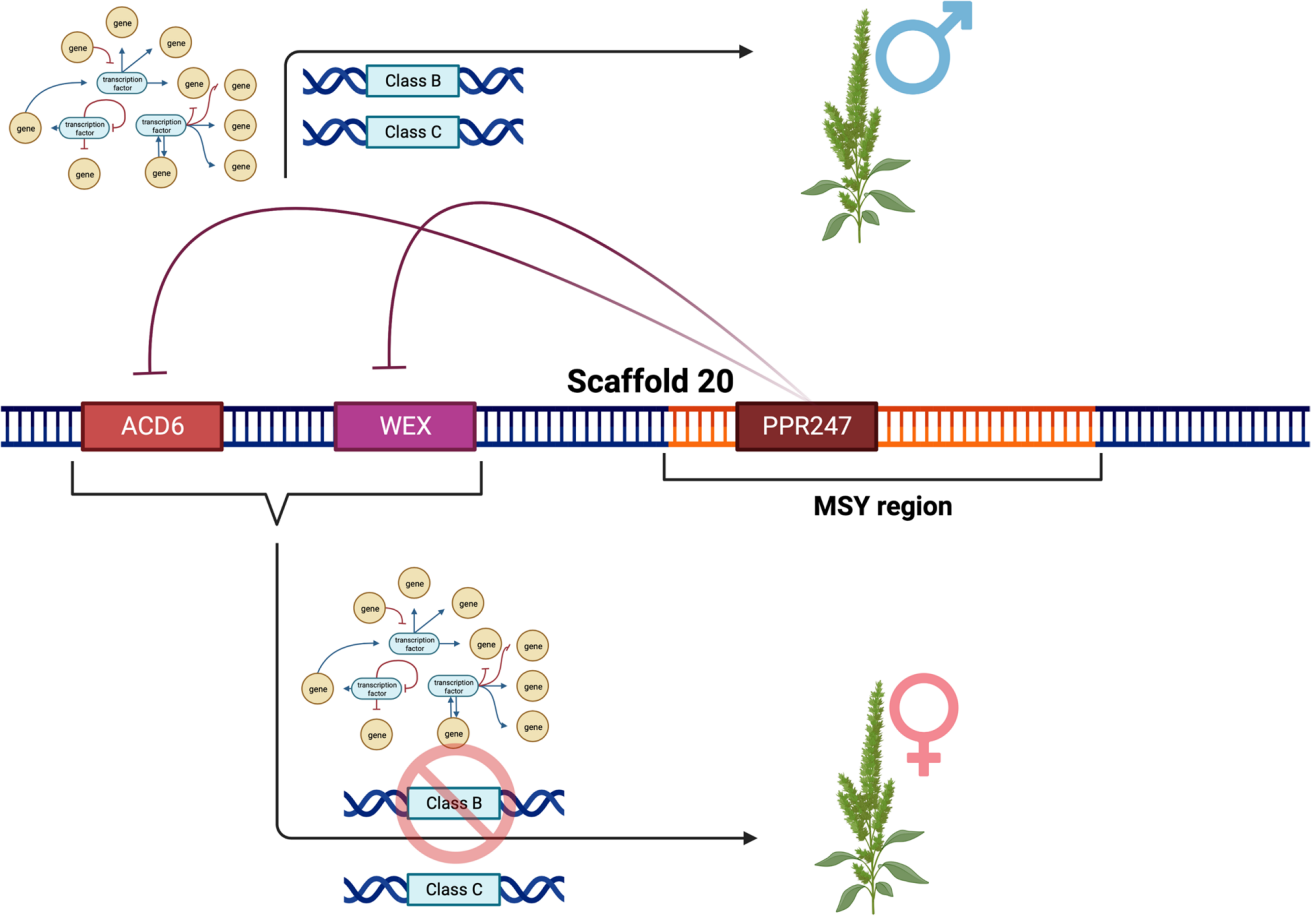
Proposed model for sex-determination in *Amaranthus palmeri*. Three genes, *PPR247*, *WEX*, and *ACD6*, play potential roles in sex-determination, with *PPR247* encoding an RNA binding protein working as a post-transcriptional repressor of *ACD6* and *WEX*, leading to a cascade of events resulting in staminate flower formation. In the absence of a male-specific region (MSY), female plants express *ACD6* and *WEX*, leading to the repression of male tissue formation via programmed cell death and epigenetic regulation of putative class B genes such as *PISTILLATA*

On the other hand, within the female tissues, two genes were uniquely expressed, *ACD6* and *WEX*, both located in scaffold 20 in linkage with the MSY region. *ACD6* is known to play a role in accelerated cell death responses, indicating that a specific programmed cell death mechanism could prevent the formation of male organs in females but is likely repressed in males due to a repressor within the MSY region. Multiple studies of the dioecy mechanism in other species show that controlled cell death can regulate sex identity [66, 126, 127]. Interestingly, previous electron microscopy work identified that developing staminate flowers contained vestigial female organs, while pistillate flowers did not have vestigial male organs [128]. The constant sex-specific expression of *ACD6* and other genes, even in SAM tissues, suggests that the production of male tissue is constantly repressed, resulting in no vestigial tissues in pistillate flowers. However, in staminate flowers, the repression of one or more repressors could lead to the formation of male tissues and the halting of pistil organs (Fig. 8). This scenario can also be translated into the ABC flowering model [129], where *ACD6* and *WEX* could work with class B repressor genes, such as AGAMOUS MADS-box encoding genes, leading to no formation of staminate tissue.

Among putative class B and C genes involved with *PPR247*, *ACD6*, and *WEX*, multiple MADS-box genes were identified as differentially expressed and co-expressed with the critical candidate genes. For instance, a gene identified as down-regulated in males within scaffold 20 was annotated as an AGAMOUS-like *AP1* gene, which is known to be essential for flower meristem identity class C genes in other species [98, 121]. Other *PISTILLATA* genes, known as class B genes, were also identified as up-regulated in male floral tissues and co-expressed with the three genes, further indicating their putative involvement in the sex-determination of *A. palmeri* [98].

Epigenetics regulatory mechanisms are also likely to play a role in sex-determination in *A. palmeri* and were already characterized as essential for general floral development in other species [73, 74]. Co-expression network analysis identified three epigenetic regulators highly co-expressed with *PPR247*: the histone acetyltransferase MYST domain and two histone methyltransferases, *ASHR1* and *ASHR2*. *MYST* regulates chromatin availability and modulates gene expression; it is also essential for gametophyte and floral tissue development [80]. *ASHR1* and *ASHR2* encode two SET domain-containing proteins involved in protein–protein interactions related to flowering time, development, and embryogenesis via the repression of floral homeotic genes such as *AGAMOUS* [101]. PPR genes are known to encode protein-RNA binding proteins indicating that they are likely to be regulating the expression of genes like *ACD6* and *WEX* via a post-transcriptional regulation [130]. These results indicate that *PPR247* is potentially involved at the top of the regulation of genes with epigenetic regulation and programmed cell death functions leading to the control of class B gene expression resulting in staminate flowers (Fig. 8).

Results also suggest that *A. palmeri* follows a single-gene model with potentially *PPR247* as the single regulator working via a post-transcriptional regulation. The best known plant XY system containing a single-gene mechanism for sex determination was found in poplar species, where a gene within the sex-determining region can suppress the expression of feminizing genes [131]. We hypothesize that in *A. palmeri, WEX* is the feminizing gene.

Other candidate genes within the *A. palmeri* autosomal part of the genome could also play a role. Multiple MADS-box genes were identified (Fig. 2). MADS-box genes are known as class B or C genes within the flowering pathway in multiple species [56]. Two MADS-box genes, *AGL11* and *AP1*, were identified with female-biased expression. Agamous-like proteins are essential for gynoecium development, and the fact that *AP1* was located within scaffold 20 also indicates that it can play a regulatory role in gynoecium flower determination. Promoter analysis identified multiple MADS-boxes motifs within DE genes’ promoter regions, indicating a likely interaction among them. A *PISTILLATA* MADS-box gene was also identified as up-regulated in *A. palmeri* males, indicating that this class B gene could also play a role in determining staminate flower formation.

The next step for confirming the hypothesis that *PPR247* is the actual sex-determinant factor for *A. palmeri,* and the role of the other identified genes, will be functional validation. Because these genes are involved with dioecy, using model plants such as *Arabidopsis* and tobacco for validation is unlikely to give the whole picture of the sex determination mechanism. The ideal scenario would be transforming the homologous species; however, no transformation protocol for *A. palmeri* is available yet, creating a demand for protocol development.

### Amaranthus tuberculatus autosomal candidate genes for sex determination

Differently from *A. palmeri,* no candidate DE gene was identified within the previously identified MSY region [51]. However, multiple candidates were identified within autosomal regions. The sex determination mechanism involving the repression of autosomal genes to define sex was already characterized within other dioecious species. For instance, persimmon (*Diospyros lotus*), which also possesses an XY system, has a mechanism where the autosomal *MALE GROWTH INHIBITOR* (*MeGI*) gene is repressed via an RNA hairpin and, through a small-RNA-based mechanism causing DNA methylation, leads to staminate flower development [132]. A similar story may be happening for *A. tuberculatus* sex-determination since the leading candidates were located in autosomal regions. Among the candidates, the most prominent one is the MADS-box *MADS18*, which was highly up-regulated in females and not detected in males. *MADS18* was identified as homologous to an agamous-like *APETALA* gene, which is known to play a role in regulating class B genes [83].

On the other hand, two *PISTILLATA* genes (*PMADS2* and *CMB2*) and the agamous-like *MADS2* were up-regulated in males (Fig. 2), indicating that they are likely working as class B genes for staminate flower formation. *PISTILLATA* genes were already classified as the central genes for floral organ identity in multiple species when associated with *APETALA3* for stamen development [121, 121]. However, it is expected that *PISTILLATA* is involved near the end of the sex-determination pathway, leading to the hypothesis that a sex-determination gene that is down-regulating *MADS18* in males causes increased expression of *PISTILLATA* genes. This scenario was already observed in other dioecious species, such as *Populus tremula* [106].

The *AGAMOUS-like MADS2* gene was co-expressed with multiple essential flowering genes, including epigenetic and post-transcriptional regulator genes, highlighting its potential function as a modulator of male organ formation. AGAMOUS-like genes are mainly involved with late male flower and pollen development, indicating that this gene is likely playing a role later in floral development [111, 133]. The AGAMOUS-like gene was also found containing a k-box domain, which was previously identified to play a critical role in the dimerization of *APETALA* and *PISTILLATA* genes to define floral organ identity [134]. Other key genes were identified, such as the *SUP* and *CLAVATA* genes, which were mainly identified as up-regulated in females. A previous study has shown that Agamous-like *SUP* and *CLAVATA* can interact to define meristem identity [101], suggesting that these genes are within the sex-determination pathway for *A. tuberculatus. SUP* was mainly expressed in female floral tissues, which was already observed in other dioecious species, such as *Silene latifolia* [102], indicating that this gene is likely essential for gynoecium flower formation.

**Fig. 9:**
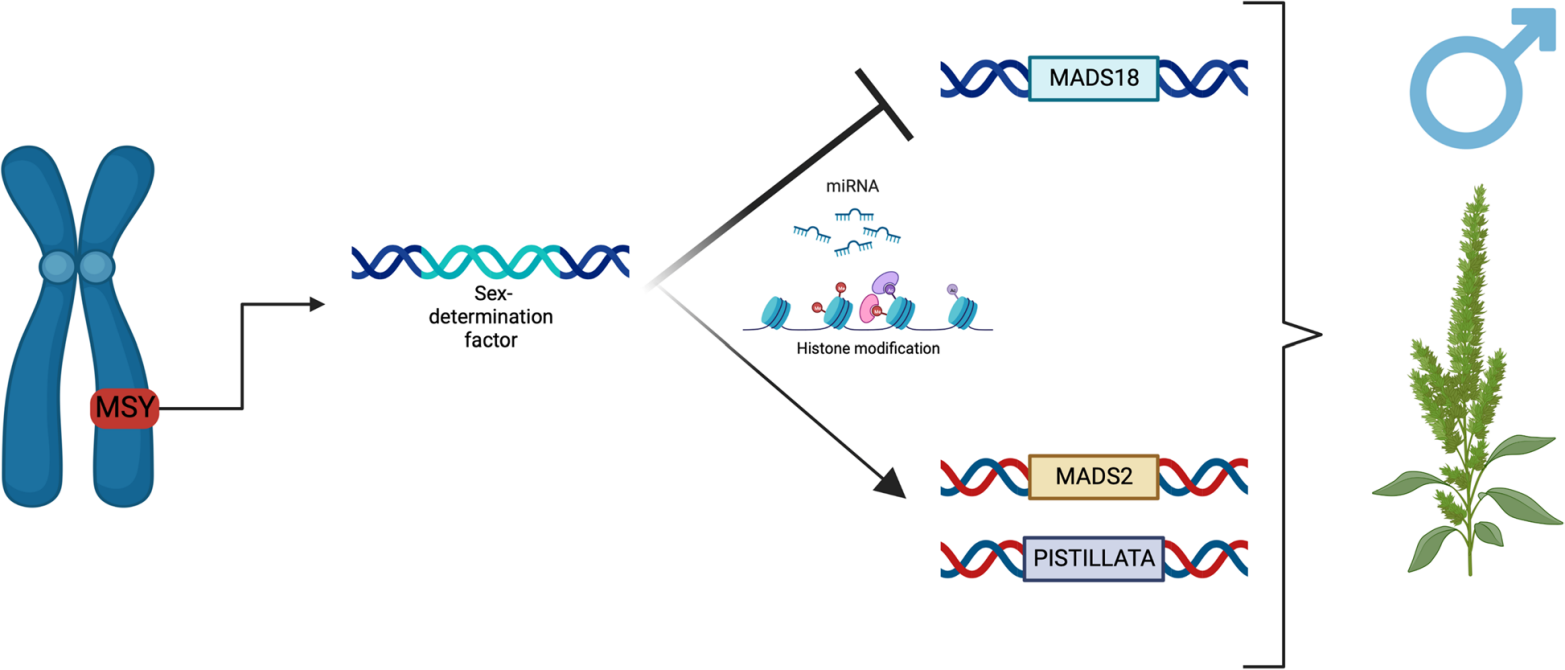
Proposed model for sex-determination in *Amaranthus tuberculatus.* A single-sex determination factor within the male-specific region (MSY) represses the feminizing *MADS18* gene, leading to the expression of crucial class B genes and inducing the production of staminate flowers. Based on gene expression results, regulation of the *PISTILLATA* and MADS-box genes is likely due to post-transcriptional regulation via miRNA silencing and epigenetic modifications

The current *A. tuberculatus* genome is still too fragmented, making the proposed MSY region a prediction of multiple contigs with putative boundary regions identified based on synteny with *A. hypochondriacus* [50]. The lack of a chromosome-level genome and a complete understanding of the MSY region makes it challenging to define whether a gene is autosomal or part of the MSY region. In fact, some of our candidates could still be within a sex-determinant region that is present in both sexes but with a different chromosomal structure, such as an inversion region. Previous studies in animals and plants have identified chromosomal inversion as an important evolutionary event for sex-determination [135, 136]. Even though multiple putative autosomal genes were identified as DE expressed across sexes, it is likely that a sex-determinant factor within the MSY region may play a role in repressing feminizing genes such as *MADS18* and inducing maleness genes such as *PISTILLATA*, leading to sex determination in *A. tuberculatus* (Fig. 9). An extensive set of DE genes involved with epigenetic regulation and post-transcriptional regulation, such as miRNA silencing, histone modification, and protein–protein interaction, were also identified, indicating that the repression of feminizing genes is likely via post-transcriptional regulation. The identity of the main sex-determination factor for *A. tuberculatus* is still unclear; however, the assembly of a chromosome-level genome may open new doors to further elucidate the sex-determination mechanism of *A. tuberculatus.* Future research will consist of the development of a chromosome level *A. tuberculatus* genome and the development of transformation protocol for subsequent gene functional validation.

### *A*. *palmeri* and *A*. *tuberculatus* have different evolution pathways for dioecy, but some essential genes are shared

Results point to a more detailed description of putative candidates for sex determination in *A. palmeri,* with a putative single-gene model located in the MSY region. However, for *A. tuberculatus*, DE genes were only identified at autosomal regions, indicating that the two species likely had different evolutionary events to dioecy. This hypothesis was previously suggested when the MSY regions from both species were identified and found to lack synteny [51]. *Amaranthus* species are known to hybridize; however, *A. palmeri* and *A. tuberculatus* do not hybridize readily, consistent with the possibility that they have distinct sex-determination mechanisms [47, 137]. Nuclear phylogenetic studies also support independent dioecy evolution in *A. tuberculatus* and *A. palmeri* in that the two species are grouped into distinct clades separated by several monoecious *Amaranthus* species [49]. The gene sequences identified in *A. palmeri* were also mapped to the *A. tuberculatus* genes to identify homologous genes. No PPR-encoding genes were DE in *A. tuberculatus* males, and no homologous gene to *PPR247* was identified in *A. tuberculatus*, indicating that this gene is unique to *A. palmeri.* Multiple copies of *WEX* and *ACD6* were identified (all located at the autosomal region) in *A. tuberculatus;* however, none were DE in the between-sex comparisons. The separate origins for dioecious and the potential involvement of *PPR247* for sex determination in *A. palmeri* are further supported by a recent genomic comparison across multiple dioecious *Amaranthus* species [138]

Major similarities between the two species that were observed in our gene-expression analyses likely were related to downstream floral development rather than the sex determination. For instance, most DE genes were predicted with similar regulations containing MYB and MADS-box motifs, indicating that those two gene families are likely involved in the floral identity pathway (Supplemental figures 15 and 16**)**. Phylogeny across DE MADS-boxes also shows that *A. tuberculatus MADS18* and *A. palmeri AP1* (Fig. 7) are highly similar, indicating homology across the two protein sequences. The expression pattern was also highly similar between the two species (Fig. 2), indicating that the AP1-like encoding gene is important for gynoecium development in both species. *PISTILLATA* DE genes were also highly conserved across the two species (Fig. 7), with *A. palmeri* showing two copies of *PISTILLATA* encoding genes, indicating that these class B genes are likely within the staminate floral determination pathway in both species.

Two other classes of genes were shared across the two species, MYB and LOB genes. MYB genes were identified as major regulators of the DE genes in both species, with the gene *MYB35* identified to be up-regulated in mature male flowers in both species. MYB TFs are critical factors in regulatory networks controlling development, metabolism, and responses to biotic and abiotic stresses [110]. The homologous gene to *MYB35* in *Arabidopsis* was previously identified to play a crucial role in anther development and was regulated by miRNA [139, 140].

Multiple LOB TFs were identified in both species, including the gene *LOB35*. LOB TFs were previously thought to only play a role in lateral organ development leading to the gene family name. However, recent studies identified that LOB TFs are involved in other functions, such as flowering control, meristematic tissue differentiation, and hormone regulation [97]. *LOB35* was identified as up-regulated in females in both species indicating a potential role in gynoecium development. A phylogeny analysis was conducted to identify the similarities across the LOB DE genes in both species. Similar to the MADS-box phylogeny, *A. palmeri* was identified with two copies of *LOB31*, while *A. tuberculatus* had only one copy, and both genes were grouped together in the phylogeny tree (Fig. 7). Among the LOBs grouped by phylogeny, only *LOB36* was highly similar between the two species and was up-regulated in males of both species.

## Conclusions

This study represents the first overall characterization of expression variation across males and females in the two major dioecious species within the *Amaranthus* genus. Key candidates were identified for *A. palmeri* sex determination, with *PPR247* hypothesized to be the key gene in a single-gene model. The sex-determination mechanism for *A. tuberculatus is* still unclear, but key autosomal genes were identified as putative players for sex determination in this dioecious species. Results strengthen the hypothesis of multiple evolutionary events for dioecy within the *Amaranthus* genus but with retained similarities across the floral development and identity pathways. Results advance our current knowledge about dioecious evolution within the *Amaranthus* genus and lead us one step further towards understanding the sex-determination mechanisms in dioecious weedy amaranths. Future studies will include the development of transformation protocols, enhancement of genomic resources, and the functional validation of the identified candidate genes.

## Methods

### Plant materials

Seeds from an *A. tuberculatus* population (GRIN ID: PI 698,378: https://npgsweb.ars-grin.gov/gringlobal/accessiondetail?id=2099571) and *A. palmeri* population (GRIN ID: PI 632,235: https://npgsweb.ars-grin.gov/gringlobal/accessiondetail?id=1031358) were used. All seeds were subjected to a 50% commercial bleach treatment for 10 min and rinsed twice with water for 10 min each. Seeds were suspended in a 0.1 g L^−1^ agarose solution and placed in a 4 C refrigerator for four weeks [141]. After stratification, seeds were germinated in Petri dishes containing blotting paper with 2.0 ml of water. Petri dishes were closed with sealing film to avoid water loss and placed in a growth chamber for 48 h set for 12/12 h day/night and 32/15 C temperature regimes.

Germinated seedlings were transplanted into plastic greenhouse flat inserts containing a soil mixture of Sunshine LC1 (Sun Gro Horticulture, Agawam, MA, USA), soil, peat, and torpedo sand (3:1:1:1 by weight) plus 13–13-13 Osmocote fertilizer (The Scotts Company, Marysville, OH, USA). Plants were grown in a growth chamber at 30/25C day/night with a 16/8-h photoperiod. At 15 days after germination (DAG), leaf tissue was collected to determine sexes using male-specific markers [50]. Tissues were harvested, placed into 1.5 mL Eppendorf tubes, and stored at –80 C. DNA was extracted using a standard CTAB buffer protocol [142]. The nucleic acid concentration was estimated by a spectrophotometer (NanoDrop 1000 Spectrophotometer, Thermo Fisher Scientific, 81 Wyman Street, Waltham, MA 02,451). At 20 DAG, four male and female plants of each species were transplanted into 3.75 L pots until flowering.

### RNA extraction and sequencing

For each sex of each species, three tissue types were collected from four biological replicates for RNA extraction (24 libraries per species): shoot apical meristem (SAM), floral meristem, and mature flowers. At 25 DAG, SAM tissue was collected, resulting in increased plant branching and subsequently generating numerous floral meristems. SAM tissues consisted of undifferentiated meristematic regions at the top of each plant. Between 50 to 60 DAG, floral meristematic tissue was collected. The remaining flowers were allowed to differentiate, and mature floral tissues were collected. Floral meristematic tissues consisted of 1–2 days of recently differentiated SAM where no sex differentiation was visible, whereas mature flowers consisted of floral tissues where differentiation between males and females was clearly visible.

Immediately after collection, all tissues were frozen in liquid nitrogen and stored at -80 C until RNA extraction. RNA was extracted using a Trizol-based method [143] with a DNase I treatment following extraction. Samples were checked for quality and quantity by running them on a Qubit analyzer, a 1% agarose gel, and a bioanalyzer. Samples were sent to the Roy J. Carver Biotechnology Center at the University of Illinois, Urbana-Champaign, for Illumina library construction and sequencing. Libraries were prepared using the Illumina TruSeq Stranded mRNAseq Sample Prep kit (Illumina Inc., San Diego, CA). 150 bp pair reads were sequenced using a NovaSeq 6000 system using one NovaSeq S4 flow cell (Supplemental table 1).

### Data pre-processing

All fastq files were filtered for low-quality reads, and adapters were trimmed using the software Trimmomatics [144]. Reads were further filtered to remove rRNA using the software SortMeRNA [145]. Two samples from mature female flowers from both species were removed due to sequencing and alignment quality issues. With the filtered data from both species and sexes, two *de-novo* genome-guided transcriptome assemblies were conducted. Reads were mapped to the *A. tuberculatus* draft genome [146] using STAR [147], and all bam files were combined using the Samtools merge tool [148]. A merged bam file was used as input in Trinity for the transcriptome assembly (Table 1) [53]. Functional annotation of the transcriptome was done using the Trinotate pipeline [149], and the final transcriptome was generated using the annotated transcripts. Both female and male reads were used in the assembly to form a comprehensive transcriptome assembly for each species. Transcriptome quality was assessed by using BUSCO [150] completeness score as done based on the viridiplantae_odb10 database, and overall quality was accessed by mapping a set of samples to it with Bowtie2 [151] and obtaining the N50 parameter using Trinity custom script (https://github.com/trinityrnaseq/trinityrnaseq/blob/master/util/TrinityStats.pl). The transcriptome was later mapped back to the genome to identify their genomic locations using GMAP [152]. All reads were pseudo-aligned to their respective assembled transcriptome using Kallisto for read counts quantification [153].

#### Principal component analysis

Principal component analysis (PCA) was conducted using the package pcaExplorer [154] to identify overall expression differences across tissues and sexes from each species. Quantified read counts from Kallisto were loaded into R and summarized to gene-level using the Tximport package [155]. The reads matrix was converted into a DESeq2 object and normalized using the variance stabilized transformation algorithm from DESeq2 [55]. Normalized reads were then loaded into pcaExplorer for analysis.

#### Differential expression analysis

Pseudo-aligned read counts were loaded into R and summarized to gene-level using Tximport. EdgeR was used to run the differential expression analysis [61]. Genes with low expression in more than 80% of samples were filtered, and the remaining genes were subjected to normalization via the trimmed mean of M-values (TMM) method. Common, trended, and tagwise dispersions were estimated using a quantile-adjusted conditional maximum likelihood. Multiple comparisons between sexes and within each tissue were conducted using a quasi-likelihood negative binomial model to account for gene-specific variability from biological and technical sources. *P*-value correction was applied using the FDR correction method. Genes were considered differentially expressed if log fold-change (logFC) was equal or above four and FDR was equal or lower than 0.05. Over-represented and conditional GO-term enrichment analyses were conducted using TopGO with the Fisher algorithm to identify the most enriched terms within the differentially expressed genes [62]. Generated gff file from GMAP was later used to identify differentially expressed genes within autosomal and the MSY regions.

#### Co-expression network analysis

A weighted co-expression network analysis (WGCNA) was conducted using the WGCNA package [78]. Gene expression counts were normalized using the DESeq2 method and converted to a log2 scale. Hierarchical clustering was used to identify outlier samples, and the constant-height tree cut function was used to remove the outliers. Soft-threshold power was estimated to approximate network topology to a free-scale model. A signed adjacency matrix was estimated via bi-weight mid-correlation followed by a signed topological overlap matrix by dissimilarity. Genes were clustered via hierarchical clustering, and the dynamic tree-cutting algorithm was used to separate genes into modules. Module eigengenes were calculated to merge similar modules and identify modules associated with sex differentiation across tissues. An intra-modular analysis was conducted to identify hub genes within modules correlated with sex differentiation. Genes were considered hubs if module membership and gene-trait significance were above 80%. Network visualization was done using Cytoscape [156].

#### Trans factor and promoter analysis

To predict the potential regulatory players for the identified differentially expressed genes, the promoter regions of those genes were extracted from the reference genomes. A promoter region was considered the 1,500 bp upstream of the transcription start site of each gene. Promoter sequences were input in PlantRegMap [92] *Arabidopsis thaliana* TFs motif databases. The regulatory prediction was made via an over-representation of TF motif enrichment (q-value <  = 0.05).

#### MADS-boxes and LOB evolutionary analysis

Coding sequences from differentially expressed MADS-boxes and LOB genes from both species' comparisons were used to predict protein sequences using TransDecoder (https://github.com/TransDecoder/TransDecoder). Protein sequences containing complete ORFs were used, and sequences containing SRF-type TF or LOB domains were used for phylogenetic comparison. Evolutionary analyses were conducted in MEGA11 [114]. The evolutionary history was inferred using the Maximum Likelihood method and Le Gascuel model [115]. The bootstrap consensus tree inferred from 500 replicates [116] represents the evolutionary history of the genes analyzed. Branches corresponding to partitions reproduced in less than 50% of bootstrap replicates were collapsed. The percentage of replicate trees in which the associated genes clustered together in the bootstrap test (500 replicates) was calculated [116]. Initial tree(s) for the heuristic search were obtained by applying Neighbor-Join and BioNJ algorithms to a matrix of pairwise distances estimated using the JTT model and then selecting the topology with a superior log likelihood value. A discrete Gamma distribution was used to model evolutionary rate differences among sites (5 categories: + G, parameter = 2.7397).

### RNA-seq validation via qPCR

To validate the differential analysis results, a set of two differentially expressed genes was tested by quantitative PCR (qPCR) in the samples used for RNA-seq analysis and an additional three individuals. The genes selected were *PMADS2-PISTILLATA* and *MYB35* because those two genes were differentially expressed in all tissues and only in mature flowers, respectively, for both species. Primers were designed to achieve an 80 to 180-bp product size and target an exon/exon junction (Supplemental table 7). Initial primer efficiency was tested by conducting a qPCR assay using a 5-step log-scale cDNA dilution. Only primer sets ranging from 95 to 100% efficiency were used. One housekeeping gene (*GAPDH*) was used as a reference for relative expression. New mature flowers, floral, and shoot apical meristem tissues were collected, and RNA was extracted as described previously. From each species, four distinct biological replicates per tissue were collected. RNA was converted to cDNA using an iScript cDNA synthesis kit (Bio-Rad Laboratories, Inc. Hercules, CA). qPCR was performed in triplicates on each sample for each primer set by combining 5ul of iTaq Universal SYBR Green Supermix (Bio-Rad Laboratories, Inc. Hercules, CA), 0.5 ul forward primer (10 uM), 0.5 ul reverse primer (10 uM), 3 ul of nuclease-free water, and 1 ul of cDNA. Relative expression was calculated using the 2–DDCt method [157] (Supplemental figure 16**)**.

## Supplementary Information

**Additional file 1: Supplemental figure 1.** Principal component analysis for Amaranthus palmeri transcriptome data.** Supplemental figure 2.** Principal component (PC) gene loading in Amaranthus palmeri for the first four primary PCs showing genes driving dispersion.** Supplemental Figure 3.** WGCNA module cluster dendrograms and soft threshold power plots were estimated for both species analysis.** Supplemental Figure 4.** Amaranthus palmeri WGCNA modules correlation.** Supplemental figure 5.** WGCNA Tan co-expression network from A. palmeri analysis. Red nodes represent the identified Hub genes within the network, and hubs were defined based on their module membership and gene-trait significance. Labels refer to Uniprot IDs from homologous annotated genes.** Supplemental Figure 6.** Amaranthus palmeri ASHR1, ASHR2 and MYST genes expression profiles.** Supplemental Figure 7.** Co-expression network from blue module key hubs from Amaranthus palmeri. Node size represents gene trait significance.** Supplemental figure 8.** Promoter analysis results from A. palmeri. Analysis conducted using genes that were differentially expressed within tissues and across genders. Promoter regions extracted from reference genome were used for the analysis. Results summary of total of transcription factors per family showing motif enrichment on the extracted promoter region.** Supplemental figure 9.** Principal component analysis for Amaranthus tuberculatus transcriptome data.** Supplemental figure 10.** Principal component (PC) gene loading in Amaranthus tuberculatus for the first four primary PCs showing genes driving dispersion.** Supplemental Figure 11.** Module correlation for Amaranthus tuberculatus WGCNA.** Supplemental Figure 12.** WGCNA palmer intra-modular analysis to identify hub genes within main modules identified in Amaranthus palmeri analysis.** Supplemental Figure 13.** WGCNA generated blue module for Amaranthus tuberculatus. Network indicates most co-expressed genes involved in putative flower development functions.** Supplemental Figure 14.** WGCNA palmer intra-modular analysis to identify hub genes within main modules identified in Amaranthus tuberculatus analysis.** Supplemental figure 15.** Promoter analysis results from A. tuberculatus. Analysis was conducted using differentially expressed genes within tissues and across genders. Promoter regions extracted from the reference genome were used for the analysis. Results summary of total of transcription factors per family showing motif enrichment on the extracted promoter region.** Supplemental Figure 16.** qPCR results for RNA-seq validation.

**Additional file 2: Supplemental table 1.** Sequenced libraries information.

**Additional file 3: Supplemental Table 2. **A. Amaranthus palmeri enriched GO terms present in DE genes for mature flower comparison within genders. B. Amaranthus palmeri between sexes mature flower comparison genes within significant Goterms. C. Genes identified uniquely in Amaranthus palmeri SAM vs floral meristem female comparison containing key enriched GO terms. D. Genes identified uniquely in Amaranthus palmeri SAM vs floral meristem male comparison containing key enriched GO terms. E. Amaranthus palmeri SAM and mature flower comparison female biased enriched GO Terms and genes in across tissue comparison. F. Amaranthus palmeri SAM and mature flower comparison female bias enriched genes. G. Amaranthus palmeri SAM and mature flower comparison male biased enriched GO Terms and genes in across tissue comparison. F. Amaranthus palmeri SAM and mature flower comparison male biased enriched genes.

**Additional file 4: Supplemental table 3. **A. Amaranthus palmeri WGCNA results: Tan module genes and their differentially expression significance. B. Amaranthus palmeri WGCNA results: Salmon module genes and their differentially expression significance. C. Amaranthus palmeri WGCNA results: Cyan module genes and their differentially expression significance. D. Amaranthus palmeri WGCNA results: Midnightblue module genes and their differentially expression significance. E. Amaranthus palmeri WGCNA results: Blue module genes and their differentially expression significance. F. Amaranthus palmeri WGCNA results: Turquoise module genes and their differentially expression significance. G. Amaranthus palmeri WGCNA results: Red module genes and their differentially expression significance.

**Additional file 5: Supplemental table 4.** A. Regulation prediction analysis results with differentially expressed genes from SAM comparison in Amaranthus palmeri. B. Regulation prediction analysis results with differentially expressed genes from Floral meristem comparison in Amaranthus palmeri. C. Regulation prediction analysis results with differentially expressed genes from mature flower comparison in Amaranthus palmeri. D. Regulation prediction analysis results with differentially expressed genes from Floral meristem comparison in Amaranthus tuberculatus. E. Regulation prediction analysis results with differentially expressed genes from mature flower comparison in Amaranthus tuberculatus. F. Transcription factor binding site prediction results using identified key genes for sex determination in Amaranthus palmeri. F. Transcription factor binding site prediction results using identified key genes for sex determination in Amaranthus tuberculatus.

**Additional file 6: Supplemental table 5.** A. Enrichment GO term analysis for genes differentially expressed in mature flower comparison across sexes in Amanrathus tuberculatus. B. Genes containing enriched GO term for genes differentially expressed in mature flower comparison across sexes in Amanrathus tuberculatus. C. Differentially expressed genes in A. tuberculatus floral meristem tissue comparison across genders. D. SAM vs Floral meristem tissue enrichment analysis results within female A. tuberculatus. E. A. tuberculatus female SAM vs Floral meristem tissue comparison enriched genes. F. SAM vs Floral meristem tissue enrichment analysis results within male A. tuberculatus. G. A. tuberculatus male SAM vs Floral meristem tissue comparison enriched genes. H. SAM vs Mature flower tissue enrichment analysis results within female A. tuberculatus. I. Female A. tuberculatus SAM vs Mature flower tissue enriched genes. J. SAM vs Mature flower tissue enrichment analysis results within male A. tuberculatus. I. Male A. tuberculatus SAM vs Mature flower tissue enriched genes.

**Additional file 7: Supplemental table 6.** A. GO enrichment analysis with Blue module in WGCNA analysis for Amaranthus tuberculatus. B. Module membership - Gene trait corralation hub genes for the blue module in WGCNA for Amaranthus tuberculatus. C. GO enrichment analysis with Yellow module in WGCNA for Amaranthus tuberculatus. D. Module membership-Gene trait corralation hub genes for the yellow module in WGCNA for Amaranthus tuberculatus. E. GO enrichment analysis with turquoise module in WGCNA for Amaranthus tuberculatus. F. Module membership-Gene trait corralation hub genes for the turquoise module in WGCNA for Amaranthus tuberculatus. G. GO enrichment analysis with brown module in WGCNA for Amaranthus tuberculatus. H. Module membership-Gene trait corralation hub genes for the brown module in WGCNA for Amaranthus tuberculatus. I. GO enrichment analysis with red module in WGCNA for Amaranthus tuberculatus. J. Module membership-Gene trait corralation hub genes for the red module in WGCNA for Amaranthus tuberculatus. K. GO enrichment analysis with green module in WGCNA for Amaranthus tuberculatus. L. Module membership-Gene trait corralation hub genes for the green module in WGCNA for Amaranthus tuberculatus.

**Additional file 8: Supplemental Table 7.** A. Primers sequences used for RNA-seq validation via qPCR.

## Data Availability

Data is available under the BioProject PRJNA934958. (Data to be released upon manuscript acceptance. Temporary link: https://dataview.ncbi.nlm.nih.gov/object/PRJNA934958?reviewer=9osu0h8dkufe49f99skmr3cd8i.)

## References

[1] Perotti VE, Larran AS, Palmieri VE, Martinatto AK, Permingeat HR (2020). Herbicide resistant weeds: a call to integrate conventional agricultural practices, molecular biology knowledge and new technologies. Plant Sci.

[2] Renton M, Chauhan BS (2017). Modelling crop-weed competition: Why, what, how and what lies ahead?. Crop Prot.

[3] Van Wychen L. Survey of the most common and troublesome weeds in broadleaf crops, fruits & vegetables in the United States and Canada. 2019. http://wssa.net/wp-content/uploads/2019-Weed-Survey_broadleaf-crops.xlsx. Accessed 10 Dec 2021.

[4] Sauer J (1955). Revision of the dioecious amaranths Madroño.

[5] Costea M, Weaver SE, Tardif FJ (2005). The biology of invasive alien plants in Canada: Amaranthus tuberculatus (Moq.) Sauer var. rudis (Sauer). Can J Plant Sci..

[6] Ward SM, Webster TM, Steckel LE (2013). Palmer amaranth (*Amaranthus palmeri*): a review. Weed Technol.

[7] Steckel LE (2007). The dioecious Amaranthus spp.: here to stay. Weed Technol.

[8] Heap I. The international survey of herbicide resistant weeds. 2023. www.weedscience.org. Accessed 18 Apr 2023.

[9] Tranel PJ (2021). Herbicide resistance in *Amaranthus tuberculatus*. Pest Manag Sci.

[10] Schwartz-Lazaro LM, Norsworthy JK, Walsh MJ, Bagavathiannan MV (2017). Efficacy of the Integrated Harrington Seed Destructor on weeds of soybean and rice production systems in the Southern United States. Crop Sci.

[11] Shergill LS, Barlow BR, Bish MD, Bradley KW (2018). Investigations of 2,4-D and multiple herbicide resistance in a Missouri waterhemp (*Amaranthus tuberculatus*) population. Weed Sci Lawrence.

[12] LeiteMontalvão AP, Kersten B, Fladung M, Müller NA (2021). The diversity and dynamics of sex determination in dioecious plants. Front Plant Sci.

[13] Renner SS (2014). The relative and absolute frequencies of angiosperm sexual systems: Dioecy, monoecy, gynodioecy, and an updated online database. Am J Bot.

[14] Aryal R, Ming R (2014). Sex determination in flowering plants: Papaya as a model system. Plant Sci.

[15] Harkess A, Huang K, van der Hulst R, Tissen B, Caplan JL, Koppula A (2020). Sex determination by two Y-linked genes in garden asparagus. Plant Cell.

[16] Sanderson BJ, Feng G, Hu N, Carlson CH, Smart LB, Keefover-Ring K (2021). Sex determination through X-Y heterogamety in *Salix nigra*. Heredity.

[17] Yu L, Ma X, Deng B, Yue J, Ming R (2021). Construction of high-density genetic maps defined sex determination region of the Y chromosome in spinach. Mol Genet Genomics.

[18] Lewis D (1942). The evolution of sex in flowering plants. Biol Rev.

[19] Mitchell CH, Diggle PK (2005). The evolution of unisexual flowers: morphological and functional convergence results from diverse developmental transitions. Am J Bot.

[20] She H, Xu Z, Zhang H, Wu J, Wang X, Liu Z (2022). Remarkable divergence of the sex-linked region between two wild spinach progenitors. Spinacia turkestanica Spinacia Tetrandra Biol.

[21] Charlesworth B, Charlesworth D (1978). A model for the evolution of dioecy and gynodioecy. Am Nat.

[22] Galfrascoli GM, Calviño A (2020). Secondary sexual dimorphism in a dioecious tree: a matter of inter-plant variability?. Flora.

[23] Adolfi A, Gantz VM, Jasinskiene N, Lee H-F, Hwang K, Terradas G (2020). Efficient population modification gene-drive rescue system in the malaria mosquito *Anopheles stephensi*. Nat Commun.

[24] Neve P (2018). Gene drive systems: do they have a place in agricultural weed management?. Pest Manag Sci.

[25] Moore RC, Harkess AE, Weingartner LA (2016). How to be a seXY plant model: A holistic view of sex-chromosome research. Botanical Soc Am.

[26] Teixeira S, Foerster K, Bernasconi G (2009). Evidence for inbreeding depression and post-pollination selection against inbreeding in the dioecious plant *Silene latifolia*. Heredity.

[27] Blackburn KB (1924). The cytological aspects of the determination of sex in the dioecious forms of *Lychnis*. Br J Exp Biol.

[28] Atanassov I, Delichère C, Filatov DA, Charlesworth D, Negrutiu I, Monéger F (2001). Analysis and evolution of two functional Y-linked loci in a plant sex chromosome system. Mol Biol Evol.

[29] Cegan R, Marais GA, Kubekova H, Blavet N, Widmer A, Vyskot B (2010). Structure and evolution of Apetala3, a sex-linked gene in *Silene latifolia*. BMC Plant Biol.

[30] Zluvova J, Nicolas M, Berger A, Negrutiu I, Monéger F (2006). Premature arrest of the male flower meristem precedes sexual dimorphism in the dioecious plant *Silene latifolia*. Proc Natl Acad Sci.

[31] Koizumi A, Yamanaka K, Nishihara K, Kazama Y, Abe T, Kawano S (2010). Two separate pathways including *SLCLV1*, *SLSTM* and *SLCUC* that control carpel development in a bisexual mutant of *Silene latifolia*. Plant Cell Physiol.

[32] Kazama Y, Nishihara K, Bergero R, Fujiwara MT, Abe T, Charlesworth D (2012). *SlWUS1*; an X-linked gene having no homologous Y-linked copy in *Silene latifolia*. G3 Genes Genomes Genet.

[33] Yang H-W, Akagi T, Kawakatsu T, Tao R (2019). Gene networks orchestrated by *MeGI*: a single-factor mechanism underlying sex determination in persimmon. Plant J.

[34] Kazama Y, Ishii K, Aonuma W, Ikeda T, Kawamoto H, Koizumi A (2016). A new physical mapping approach refines the sex-determining gene positions on the *Silene latifolia* Y-chromosome. Sci Rep.

[35] Krasovec M, Zhang Y, Filatov DA (2020). The location of the pseudoautosomal boundary in *Silene latifolia*. Genes.

[36] Kazama Y, Kitoh M, Kobayashi T, Ishii K, Krasovec M, Yasui Y (2022). A *CLAVATA3*-like gene acts as a gynoecium suppression function in White Campion. Mol Biol Evol.

[37] VanBuren R, Zeng F, Chen C, Zhang J, Wai CM, Han J (2015). Origin and domestication of papaya Y^h^ chromosome. Genome Res.

[38] Akagi T, Pilkington SM, Varkonyi-Gasic E, Henry IM, Sugano SS, Sonoda M (2019). Two Y-chromosome-encoded genes determine sex in kiwifruit. Nat Plants.

[39] Akagi T, Henry IM, Ohtani H, Morimoto T, Beppu K, Kataoka I (2018). A Y-encoded suppressor of feminization arose via lineage-specific duplication of a cytokinin response regulator in kiwifruit. Plant Cell.

[40] Varkonyi-Gasic E, Wang T, Cooney J, Jeon S, Voogd C, Douglas MJ (2021). *Shy Girl*, a kiwifruit suppressor of feminization, restricts gynoecium development via regulation of cytokinin metabolism and signalling. New Phytol.

[41] Bachtrog D, Mank JE, Peichel CL, Kirkpatrick M, Otto SP, Ashman T-L (2014). Sex determination: why so many ways of doing it?. PLoS Biol.

[42] Henry IM, Akagi T, Tao R, Comai L (2018). One hundred ways to invent the sexes: theoretical and observed paths to dioecy in plants. Annu Rev Plant Biol.

[43] Goldberg EE, Otto SP, Vamosi JC, Mayrose I, Sabath N, Ming R (2017). Macroevolutionary synthesis of flowering plant sexual systems. Evolution.

[44] Geraldes A, Hefer CA, Capron A, Kolosova N, Martinez-Nuñez F, Soolanayakanahally RY (2015). Recent Y chromosome divergence despite ancient origin of dioecy in poplars (*Populus*). Mol Ecol.

[45] Kersten B, Pakull B, Groppe K, Lueneburg J, Fladung M (2014). The sex-linked region in Populus tremuloides Turesson 141 corresponds to a pericentromeric region of about two million base pairs on *P. *trichocarpa chromosome 19. Plant Biol.

[46] Paolucci I, Gaudet M, Jorge V, Beritognolo I, Terzoli S, Kuzminsky E (2010). Genetic linkage maps of Populus alba L. and comparative mapping analysis of sex determination across Populus species. Tree Genet Genomes.

[47] Murray MJ (1940). The genetics of sex determination in the family *Amaranthaceae*. Genetics.

[48] Grant WF (1959). Cytogenetic studies in Amaranthus: II. natural interspecific hybridization between Amaranthus dubius and A. spinosus. Can J Bot..

[49] Waselkov KE, Boleda AS, Olsen KM (2018). A phylogeny of the genus *Amaranthus* (*Amaranthaceae*) based on several low-copy nuclear loci and chloroplast regions. Syst Bot.

[50] Montgomery JS, Sadeque A, Giacomini DA, Brown PJ, Tranel PJ (2019). Sex-specific markers for waterhemp (*Amaranthus tuberculatus*) and Palmer amaranth (*Amaranthus palmeri*). Weed Sci.

[51] Montgomery JS, Giacomini DA, Weigel D, Tranel PJ (2021). Male-specific Y-chromosomal regions in waterhemp (*Amaranthus tuberculatus*) and Palmer amaranth (*Amaranthus palmeri*). New Phytol.

[52] Neves CJ, Matzrafi M, Thiele M, Lorant A, Mesgaran MB, Stetter MG (2020). Male linked genomic regions determine sex in dioecious *Amaranthus palmeri*. J Hered.

[53] Haas BJ, Papanicolaou A, Yassour M, Grabherr M, Blood PD, Bowden J (2013). De novo transcript sequence reconstruction from RNA-seq using the Trinity platform for reference generation and analysis. Nat Protoc.

[54] Bray NL, Pimentel H, Melsted P, Pachter L (2016). Near-optimal probabilistic RNA-seq quantification. Nat Biotechnol.

[55] Love MI, Huber W, Anders S (2014). Moderated estimation of fold change and dispersion for RNA-seq data with DESeq2. Genome Biol.

[56] Lee H, Suh S-S, Park E, Cho E, Ahn JH, Kim S-G (2000). The *AGAMOUS-LIKE 20* MADS domain protein integrates floral inductive pathways in *Arabidopsis*. Genes Dev.

[57] Lee J, Oh M, Park H, Lee I (2008). *SOC1* translocated to the nucleus by interaction with *AGL24* directly regulates *LEAFY*. Plant J.

[58] Escobar-Restrepo J-M, Huck N, Kessler S, Gagliardini V, Gheyselinck J, Yang W-C (2007). The *FERONIA* receptor-like kinase mediates male-female interactions during pollen tube reception. Science.

[59] Wu M-F, Sang Y, Bezhani S, Yamaguchi N, Han S-K, Li Z (2012). *SWI2/SNF2* chromatin remodeling ATPases overcome polycomb repression and control floral organ identity with the *LEAFY* and *SEPALLATA3* transcription factors. Proc Natl Acad Sci.

[60] Gan E-S, Huang J, Ito T (2013). Functional roles of histone modification, chromatin remodeling and microRNAs in *Arabidopsis* flower development. Int Rev Cell Mol Biol.

[61] Robinson MD, McCarthy DJ, Smyth GK (2010). EdgeR: a Bioconductor package for differential expression analysis of digital gene expression data. Bioinformatics.

[62] Alexa A, Rahnenführer J (2009). Gene set enrichment analysis with topGO. Bioconductor Improv.

[63] Vizcay-Barrena G, Wilson ZA (2006). Altered tapetal PCD and pollen wall development in the *Arabidopsis* ms1 mutant. J Exp Bot.

[64] Wilson ZA, Morroll SM, Dawson J, Swarup R, Tighe PJ (2001). The Arabidopsis *MALE STERILITY1 (MS1)* gene is a transcriptional regulator of male gametogenesis, with homology to the PHD-finger family of transcription factors. Plant J.

[65] Aya K, Ueguchi-Tanaka M, Kondo M, Hamada K, Yano K, Nishimura M (2009). Gibberellin modulates anther development in rice via the transcriptional regulation of *GAMYB*. Plant Cell.

[66] Phan HA, Iacuone S, Li SF, Parish RW (2011). The *MYB80* transcription factor is required for pollen development and the regulation of tapetal programmed cell death in *Arabidopsis thaliana*. Plant Cell.

[67] Groszmann M, Paicu T, Smyth DR (2008). Functional domains of *SPATULA*, a bHLH transcription factor involved in carpel and fruit development in *Arabidopsis*. Plant J.

[68] Zhang W, Sun Y, Timofejeva L, Chen C, Grossniklaus U, Ma H (2006). Regulation of *Arabidopsis* tapetum development and function by *DYSFUNCTIONAL TAPETUM1 (DYT1)* encoding a putative bHLH transcription factor. Development.

[69] Kuusk S, Sohlberg JJ, Magnus Eklund D, Sundberg E (2006). Functionally redundant *SHI* family genes regulate *Arabidopsis* gynoecium development in a dose-dependent manner. Plant J.

[70] Yoo SK, Lee JS, Ahn JH (2006). Overexpression of *AGAMOUS-LIKE 28 (AGL28)* promotes flowering by upregulating expression of floral promoters within the autonomous pathway. Biochem Biophys Res Commun.

[71] Somssich M, Je BI, Simon R, Jackson D (2016). *CLAVATA-WUSCHEL* signaling in the shoot meristem. Development.

[72] Aukerman MJ, Sakai H (2003). Regulation of flowering time and floral organ identity by a microrna and its *APETALA2-like* target genes. Plant Cell.

[73] Gady ALF, Alves CS, Nogueira FTS, Rajewsky N, Jurga S, Barciszewski J (2017). Epigenetics in plant reproductive development: an overview from flowers to seeds. Plant Epigenetics.

[74] Bräutigam K, Soolanayakanahally R, Champigny M, Mansfield S, Douglas C, Campbell MM (2017). Sexual epigenetics: gender-specific methylation of a gene in the sex determining region of *Populus balsamifera*. Sci Rep.

[75] Klucher KM, Chow H, Reiser L, Fischer RL (1996). The *AINTEGUMENTA* gene of *Arabidopsis* required for ovule and female gametophyte development is related to the floral homeotic gene *APETALA2*. Plant Cell.

[76] Krizek BA, Bantle AT, Heflin JM, Han H, Freese NH, Loraine AE (2021). *AINTEGUMENTA* and *AINTEGUMENTA-LIKE6* directly regulate floral homeotic, growth, and vascular development genes in young *Arabidopsis* flowers. J Exp Bot.

[77] Krizek BA, Blakley IC, Ho Y-Y, Freese N, Loraine AE (2020). The *Arabidopsis* transcription factor *AINTEGUMENTA* orchestrates patterning genes and auxin signaling in the establishment of floral growth and form. Plant J.

[78] Langfelder P, Horvath S (2008). WGCNA: an R package for weighted correlation network analysis. BMC Bioinformatics.

[79] Xiao J, Zhang H, Xing L, Xu S, Liu H, Chong K (2013). Requirement of histone acetyltransferases *HAM1* and *HAM2* for epigenetic modification of *FLC* in regulating flowering in *Arabidopsis*. J Plant Physiol.

[80] Latrasse D, Benhamed M, Henry Y, Domenichini S, Kim W, Zhou D-X (2008). The *MYST* histone acetyltransferases are essential for gametophyte development in *Arabidopsis*. BMC Plant Biol.

[81] Grini PE, Thorstensen T, Alm V, Vizcay-Barrena G, Windju SS, Jørstad TS (2009). The *ASH1 homolog 2 (ASHH2)* histone H3 methyltransferase is required for ovule and anther development in *Arabidopsis*. PLoS ONE.

[82] Sreekantan L, Torregrosa L, Fernandez L, Thomas MR (2006). *VvMADS9*, a class B MADS-box gene involved in grapevine flowering, shows different expression patterns in mutants with abnormal petal and stamen structures. Funct Plant Biol.

[83] Krogan NT, Hogan K, Long JA (2012). *APETALA2* negatively regulates multiple floral organ identity genes in *Arabidopsis* by recruiting the co-repressor *TOPLESS* and the histone deacetylase *HDA19*. Development.

[84] Kitomi Y, Ito H, Hobo T, Aya K, Kitano H, Inukai Y (2011). The auxin responsive AP2/ERF transcription factor *CROWN ROOTLESS5* is involved in crown root initiation in rice through the induction of *OsRR1*, a type-A response regulator of cytokinin signaling. Plant J.

[85] Western TL, Cheng Y, Liu J, Chen X (2002). *HUA ENHANCER2*, a putative DExH-box RNA helicase, maintains homeotic B and C gene expression in *Arabidopsis*. Development.

[86] Mockler TC, Yu X, Shalitin D, Parikh D, Michael TP, Liou J (2004). Regulation of flowering time in *Arabidopsis* by K homology domain proteins. Proc Natl Acad Sci U S A.

[87] Ortuño-Miquel S, Rodríguez-Cazorla E, Zavala-Gonzalez EA, Martínez-Laborda A, Vera A (2019). *Arabidopsis HUA ENHANCER 4* delays flowering by upregulating the MADS-box repressor genes *FLC* and *MAF4*. Sci Rep.

[88] Chen X, Liu J, Cheng Y, Jia D (2002). *HEN1* functions pleiotropically in *Arabidopsis* development and acts in C function in the flower. Development.

[89] Park W, Li J, Song R, Messing J, Chen X (2002). *CARPEL FACTORY*, a Dicer homolog, and *HEN1*, a novel protein, act in microRNA metabolism in *Arabidopsis thaliana*. Curr Biol.

[90] Wang T, Ping X, Cao Y, Jian H, Gao Y, Wang J (2019). Genome-wide exploration and characterization of miR172/euAP2 genes in Brassica napus L. for likely role in flower organ development. BMC Plant Biol.

[91] Wei S-J, Chai S, Zhu R-M, Duan C-Y, Zhang Y, Li S (2020). *HUA ENHANCER1* mediates ovule development. Front Plant Sci..

[92] Tian F, Yang D-C, Meng Y-Q, Jin J, Gao G (2020). PlantRegMap: charting functional regulatory maps in plants. Nucleic Acids Res.

[93] Andersen SU, Algreen-Petersen RG, Hoedl M, Jurkiewicz A, Cvitanich C, Braunschweig U (2007). The conserved cysteine-rich domain of a tesmin/TSO1-like protein binds zinc in vitro and *TSO1* is required for both male and female fertility in *Arabidopsis thaliana*. J Exp Bot.

[94] Feng K, Hou X-L, Xing G-M, Liu J-X, Duan A-Q, Xu Z-S (2020). Advances in AP2/ERF super-family transcription factors in plant. Crit Rev Biotechnol.

[95] Alvarez-Buylla ER, Liljegren SJ, Pelaz S, Gold SE, Burgeff C, Ditta GS (2000). MADS-box gene evolution beyond flowers: expression in pollen, endosperm, guard cells, roots and trichomes. Plant J.

[96] Fornara F, Pařenicová L, Falasca G, Pelucchi N, Masiero S, Ciannamea S (2004). Functional characterization of *OsMADS18*, a member of the AP1/SQUA subfamily of MADS box genes. Plant Physiol.

[97] Zhang Y, Li Z, Ma B, Hou Q, Wan X (2020). Phylogeny and functions of LOB domain proteins in plants. Int J Mol Sci.

[98] Poupin MJ, Federici F, Medina C, Matus JT, Timmermann T, Arce-Johnson P (2007). Isolation of the three grape sub-lineages of B-class MADS-box *TM6 PISTILLATA* and *APETALA3* genes which are differentially expressed during flower and fruit development. Gene.

[99] Immink RGH, Ferrario S, Busscher-Lange J, Kooiker M, Busscher M, Angenent GC (2003). Analysis of the petunia MADS-box transcription factor family. Mol Genet Genomics.

[100] Sakai H, Krizek BA, Jacobsen SE, Meyerowitz EM (2000). Regulation of *SUP* expression identifies multiple regulators involved in Arabidopsis floral meristem development. Plant Cell.

[101] Uemura A, Yamaguchi N, Xu Y, Wee W, Ichihashi Y, Suzuki T (2018). Regulation of floral meristem activity through the interaction of *AGAMOUS*, *SUPERMAN*, and *CLAVATA3* in *Arabidopsis*. Plant Reprod.

[102] Kazama Y, Fujiwara MT, Koizumi A, Nishihara K, Nishiyama R, Kifune E (2009). A SUPERMAN-like gene is exclusively expressed in female flowers of the dioecious plant *Silene latifolia*. Plant Cell Physiol.

[103] Song Y, Ma K, Ci D, Tian X, Zhang Z, Zhang D (2013). The *SUPERMAN* gene family in *Populus*: nucleotide diversity and gene expression in a dioecious plant. Plant Cell Rep.

[104] Jack T (2002). New members of the floral organ identity *AGAMOUS* pathway. Trends Plant Sci.

[105] Samach A, Klenz JE, Kohalmi SE, Risseeuw E, Haughn GW, Crosby WL (1999). The *UNUSUAL FLORAL ORGANS* gene of *Arabidopsis thaliana* is an F-box protein required for normal patterning and growth in the floral meristem. Plant J.

[106] Leite Montalvão AP, Kersten B, Kim G, Fladung M, Müller NA (2022). *ARR17* controls dioecy in Populus by repressing B-class MADS-box gene expression. Philos Trans R Soc B.

[107] Koyama T, Furutani M, Tasaka M, Ohme-Takagi M (2007). TCP transcription factors control the morphology of shoot lateral organs via negative regulation of the expression of boundary-specific genes in *Arabidopsis*. Plant Cell.

[108] Dinneny JR, Weigel D, Yanofsky MF (2006). *NUBBIN* and *JAGGED* define stamen and carpel shape in *Arabidopsis*. Development.

[109] Xu B, Li Z, Zhu Y, Wang H, Ma H, Dong A (2008). *Arabidopsis* genes *AS1*, *AS2*, and *JAG* negatively regulate boundary-specifying genes to promote sepal and petal development. Plant Physiol.

[110] Dubos C, Stracke R, Grotewold E, Weisshaar B, Martin C, Lepiniec L (2010). MYB transcription factors in *Arabidopsis*. Trends Plant Sci.

[111] Hsu W-H, Yeh T-J, Huang K-Y, Li J-Y, Chen H-Y, Yang C-H (2014). *AGAMOUS-LIKE13*, a putative ancestor for the E functional genes, specifies male and female gametophyte morphogenesis. Plant J Cell Mol Biol.

[112] Xu Z-S, Chen M, Li L-C, Ma Y-Z (2008). Functions of the ERF transcription factor family in plants. Botany.

[113] Mitsuda N, Ohme-Takagi M (2009). Functional analysis of transcription factors in *Arabidopsis*. Plant Cell Physiol.

[114] Tamura K, Stecher G, Kumar S (2021). MEGA11: molecular evolutionary genetics analysis version 11. Mol Biol Evol.

[115] Le SQ, Gascuel O (2008). An improved general amino acid replacement matrix. Mol Biol Evol.

[116] Felsenstein J (1985). Confidence limits on phylogenies: an approach using the bootstrap. Evolution.

[117] Yu H, Xu Y, Tan EL, Kumar PP (2002). GAMOUS-LIKE 24, a dosage-dependent mediator of the flowering signals. Proc Natl Acad Sci.

[118] Michaels SD, Ditta G, Gustafson-Brown C, Pelaz S, Yanofsky M, Amasino RM (2003). *AGL24* acts as a promoter of flowering in *Arabidopsis* and is positively regulated by vernalization. Plant J.

[119] Liu C, Zhou J, Bracha-Drori K, Yalovsky S, Ito T, Yu H (2007). Specification of Arabidopsis floral meristem identity by repression of flowering time genes. Development.

[120] Royo C, Torres-Pérez R, Mauri N, Diestro N, Cabezas JA, Marchal C (2018). The major origin of seedless grapes is associated with a missense mutation in the mads-box gene *VviAGL11*. Plant Physiol.

[121] Pfent C, Pobursky KJ, Sather DN, Golenberg EM (2005). Characterization of *SpAPETALA3* and *SpPISTILLATA*, B class floral identity genes in *Spinacia oleracea*, and their relationship to sexual dimorphism. Dev Genes Evol.

[122] Honma T, Goto K (2000). The *Arabidopsis* floral homeotic gene *PISTILLATA* is regulated by discrete cis-elements responsive to induction and maintenance signals. Development.

[123] Barrett SCH (1998). The evolution of mating strategies in flowering plants. Trends Plant Sci.

[124] Bentolila S, Alfonso AA, Hanson MR (2002). A pentatricopeptide repeat-containing gene restores fertility to cytoplasmic male-sterile plants. Proc Natl Acad Sci.

[125] Wang Y, Hou Y, Gu H, Kang D, Chen Z, Liu J (2012). The *Arabidopsis APC4* subunit of the anaphase-promoting complex/cyclosome (APC/C) is critical for both female gametogenesis and embryogenesis. Plant J.

[126] Coimbra S, Torrão L, Abreu I (2004). Programmed cell death induces male sterility in *Actinidia deliciosa* female flowers. Plant Physiol Biochem.

[127] Flores-Rentería L, Orozco-Arroyo G, Cruz-García F, García-Campusano F, Alfaro I, Vázquez-Santana S (2013). Programmed cell death promotes male sterility in the functional dioecious *Opuntia stenopetala* (*Cactaceae*). Ann Bot.

[128] Wenzhuo W, Jernstedt J, Mesgaran M. The comparative flower development of Palmer amaranth: male vs. female. In: 2022 Proceedings of the California Weed Science Society: 19-21 January 2022 Sacramento, California. Edited by Anil Shrestha; 2022. p. 46.

[129] Bowman JL, Smyth DR, Meyerowitz EM (2012). The ABC model of flower development: then and now. Development.

[130] Saha D, Prasad AM, Srinivasan R (2007). Pentatricopeptide repeat proteins and their emerging roles in plants. Plant Physiol Biochem.

[131] Müller NA, Kersten B, Leite Montalvão AP, Mähler N, Bernhardsson C, Bräutigam K (2020). A single gene underlies the dynamic evolution of poplar sex determination. Nat Plants.

[132] Akagi T, Henry IM, Tao R, Comai L (2014). A Y-chromosome–encoded small RNA acts as a sex determinant in persimmons. Science.

[133] Theissen G, Becker A, Di Rosa A, Kanno A, Kim JT, Münster T (2000). A short history of MADS-box genes in plants. Plant Mol Biol.

[134] Yang Y, Fanning L, Jack T (2003). The K domain mediates heterodimerization of the *Arabidopsis* floral organ identity proteins, *APETALA3* and *PISTILLATA*. Plant J.

[135] Zhang J, Boualem A, Bendahmane A, Ming R (2014). Genomics of sex determination. Curr Opin Plant Biol.

[136] Natri HM, Merilä J, Shikano T (2019). The evolution of sex determination associated with a chromosomal inversion. Nat Commun.

[137] Trucco F, Zheng D, Woodyard AJ, Walter JR, Tatum TC, Rayburn AL (2007). Nonhybrid progeny from crosses of dioecious amaranths: implications for gene-flow research. Weed Sci.

[138] Raiyemo DA, Bobadilla LK, Tranel PJ (2023). Genomic profiling of dioecious Amaranthus species provides novel insights into species relatedness and sex genes. BMC Biol.

[139] Allen RS, Li J, Stahle MI, Dubroué A, Gubler F, Millar AA (2007). Genetic analysis reveals functional redundancy and the major target genes of the *Arabidopsis* miR159 family. Proc Natl Acad Sci.

[140] Zhang Z-B, Zhu J, Gao J-F, Wang C, Li H, Li H (2007). Transcription factor *AtMYB103* is required for anther development by regulating tapetum development, callose dissolution and exine formation in *Arabidopsis*. Plant J.

[141] Bell MS, Hager AG, Tranel PJ (2013). Multiple resistance to herbicides from four site-of-action groups in waterhemp (*Amaranthus tuberculatus*). Weed Sci.

[142] Porebski S, Bailey LG, Baum BR (1997). Modification of a CTAB DNA extraction protocol for plants containing high polysaccharide and polyphenol components. Plant Mol Biol Report.

[143] Simms D, Cizdziel PE, Chomczynski P (1993). TRIzol: A new reagent for optimal single-step isolation of RNA. Focus.

[144] Bolger AM, Lohse M, Usadel B (2014). Trimmomatic: a flexible trimmer for Illumina sequence data. Bioinformatics.

[145] Kopylova E, Noé L, Touzet H (2012). SortMeRNA: fast and accurate filtering of ribosomal RNAs in metatranscriptomic data. Bioinformatics.

[146] Montgomery JS, Giacomini D, Waithaka B, Lanz C, Murphy BP, Campe R (2020). Draft Genomes of *Amaranthus tuberculatus*, *Amaranthus hybridus*, and *Amaranthus palmeri*. Genome Biol Evol.

[147] Dobin A, Gingeras TR (2015). Mapping RNA-seq reads with STAR. Curr Protoc Bioinforma.

[148] Li H, Handsaker B, Wysoker A, Fennell T, Ruan J, Homer N (2009). The sequence alignment/map format and SAMtools. Bioinformatics.

[149] Bryant DM, Johnson K, DiTommaso T, Tickle T, Couger MB, Payzin-Dogru D (2017). A tissue-mapped axolotl de novo transcriptome enables identification of limb regeneration factors. Cell Rep.

[150] Simão FA, Waterhouse RM, Ioannidis P, Kriventseva EV, Zdobnov EM (2015). BUSCO: assessing genome assembly and annotation completeness with single-copy orthologs. Bioinformatics.

[151] Langdon WB (2015). Performance of genetic programming optimised Bowtie2 on genome comparison and analytic testing (GCAT) benchmarks. BioData Min.

[152] Wu TD, Watanabe CK (2005). GMAP: a genomic mapping and alignment program for mRNA and EST sequences. Bioinformatics.

[153] Pimentel H, Bray NL, Puente S, Melsted P, Pachter L (2017). Differential analysis of RNA-seq incorporating quantification uncertainty. Nat Methods.

[154] Marini F, Binder H (2019). pcaExplorer: an R/Bioconductor package for interacting with RNA-seq principal components. BMC Bioinformatics.

[155] Love MI, Soneson C, Robinson MD (2017). Importing transcript abundance datasets with tximport. Dim Txi Inf Rep Sample1.

[156] Kohl M, Wiese S, Warscheid B, Hamacher M, Eisenacher M, Stephan C (2011). Cytoscape: Software for Visualization and Analysis of Biological Networks. Data Mining in Proteomics: From Standards to Applications.

[157] Livak KJ, Schmittgen TD (2001). Analysis of relative gene expression data using real-time quantitative PCR and the 2- ΔΔCT method. Methods.

