# Supplementary material for: Comparative transcriptomic analysis of male and females in the dioecious weeds Amaranthus palmeri and Amaranthus tuberculatus
Source: BMC Plant Biol. 2023 Jun 26;23:339. doi: 10.1186/s12870-023-04286-9 (PMC10291800; doi:10.1186/s12870-023-04286-9)
Supplement: Supplementary file 1 — Additional file 1: Supplemental figure 1. Principal component analysis for Amaranthus palmeri transcriptome data. Supplemental figure 2. Principal component (PC) gene loading in Amaranthus palmeri for the first four primary PCs showing genes driving dispersion. Supplemental Figure 3. WGCNA module cluster dendrograms and soft threshold power plots were estimated for both species analysis. Supplemental Figure 4. Amaranthus palmeri WGCNA modules correlation. Supplemental figure 5. WGCNA Tan co-expression network from A. palmeri analysis. Red nodes represent the identified Hub genes within the network, and hubs were defined based on their module membership and gene-trait significance. Labels refer to Uniprot IDs from homologous annotated genes. Supplemental Figure 6. Amaranthus palmeri ASHR1, ASHR2 and MYST genes expression profiles. Supplemental Figure 7. Co-expression network from blue module key hubs from Amaranthus palmeri. Node size represents gene trait significance. Supplemental figure 8. Promoter analysis results from A. palmeri. Analysis conducted using genes that were differentially expressed within tissues and across genders. Promoter regions extracted from reference genome were used for the analysis. Results summary of total of transcription factors per family showing motif enrichment on the extracted promoter region. Supplemental figure 9. Principal component analysis for Amaranthus tuberculatus transcriptome data. Supplemental figure 10. Principal component (PC) gene loading in Amaranthus tuberculatus for the first four primary PCs showing genes driving dispersion. Supplemental Figure 11. Module correlation for Amaranthus tuberculatus WGCNA. Supplemental Figure 12. WGCNA palmer intra-modular analysis to identify hub genes within main modules identified in Amaranthus palmeri analysis. Supplemental Figure 13. WGCNA generated blue module for Amaranthus tuberculatus. Network indicates most co-expressed genes involved in putative flower development functions. S [file 12870_2023_4286_MOESM1_ESM.docx]

Supplemental figures

Supplemental figure 1. Principal component analysis for Amaranthus palmeri transcriptome data.

Supplemental figure 2. Principal component (PC) gene loading in Amaranthus palmeri for the first four primary PCs showing genes driving dispersion.


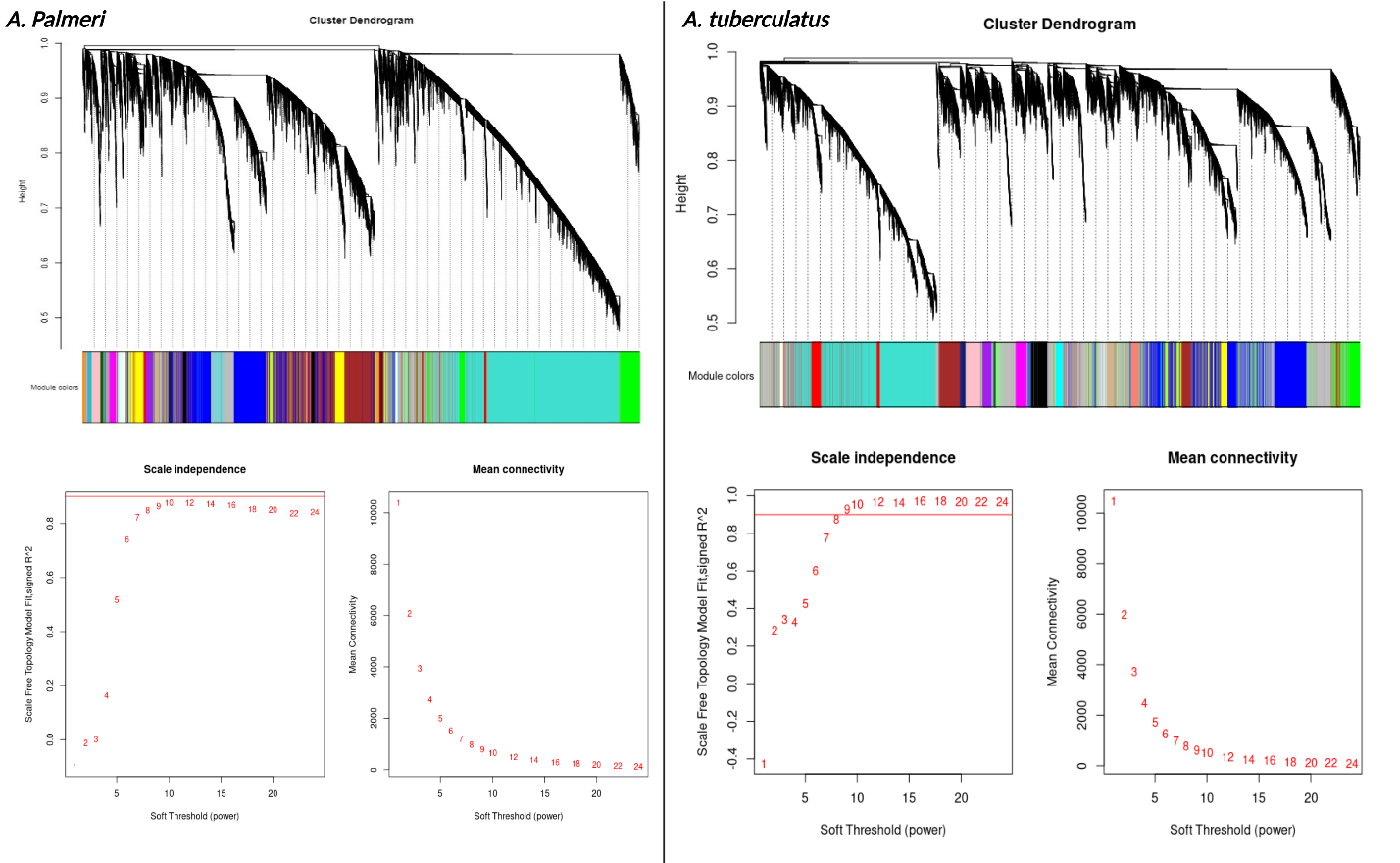


Supplemental Figure 3. WGCNA module cluster dendrograms and soft threshold power plots were estimated for both species analysis.


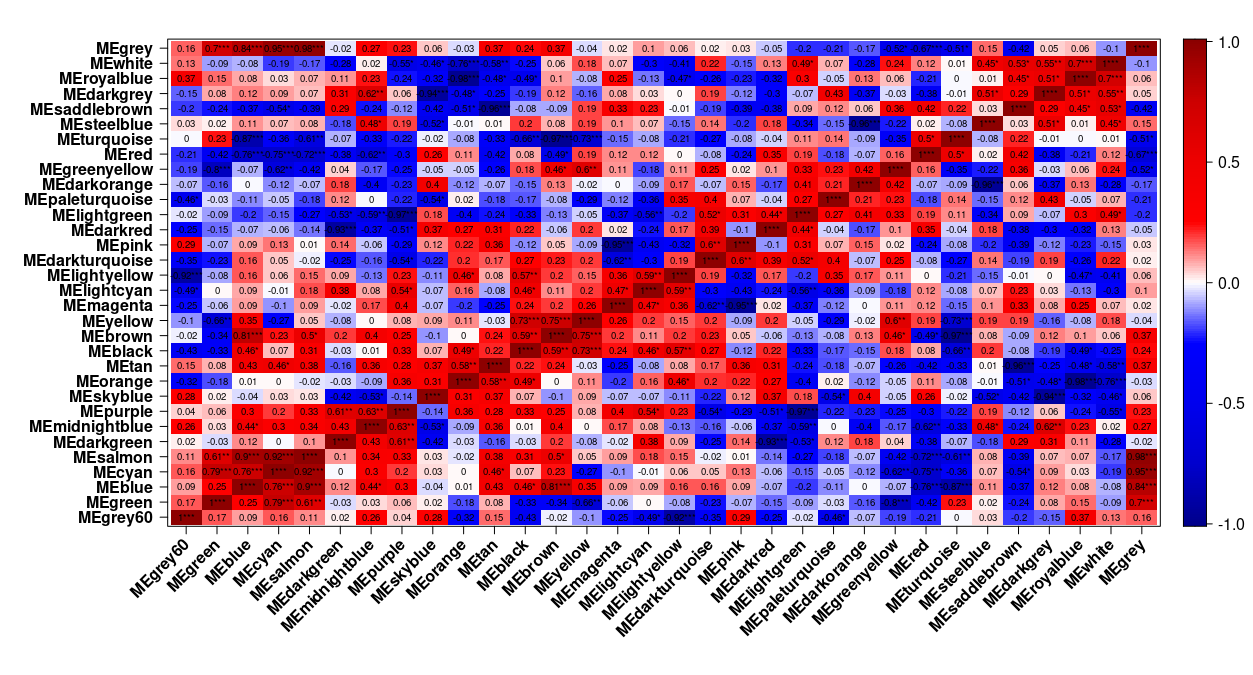


Supplemental Figure 4. Amaranthus palmeri WGCNA modules correlation

Supplemental figure 5. WGCNA Tan co-expression network from A. palmeri analysis. Red nodes represent the identified Hub genes within the network, and hubs were defined based on their module membership and gene-trait significance. Labels refer to Uniprot IDs from homologous annotated genes.


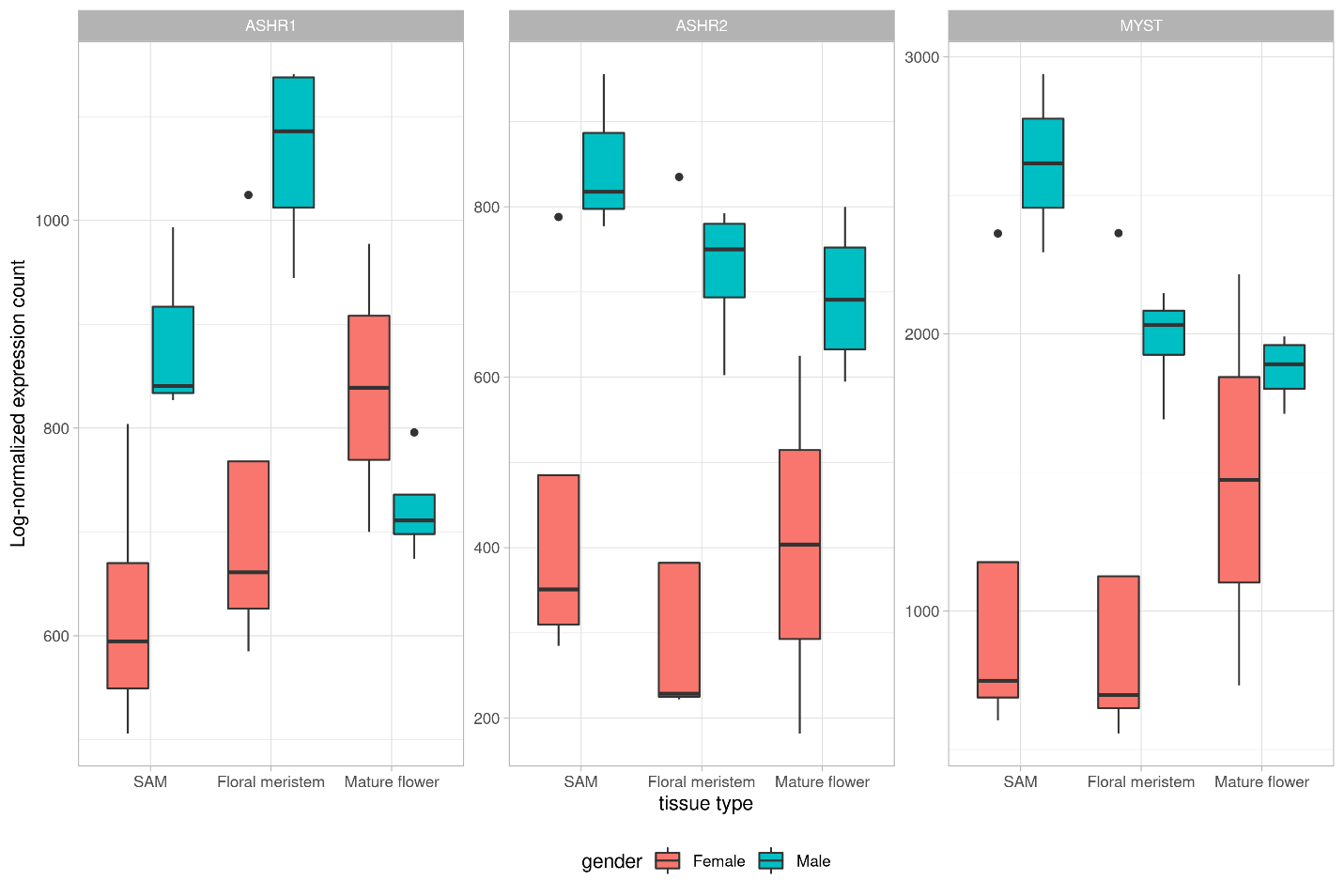


Supplemental Figure 6. Amaranthus palmeri ASHR1, ASHR2 and MYST genes expression profiles.


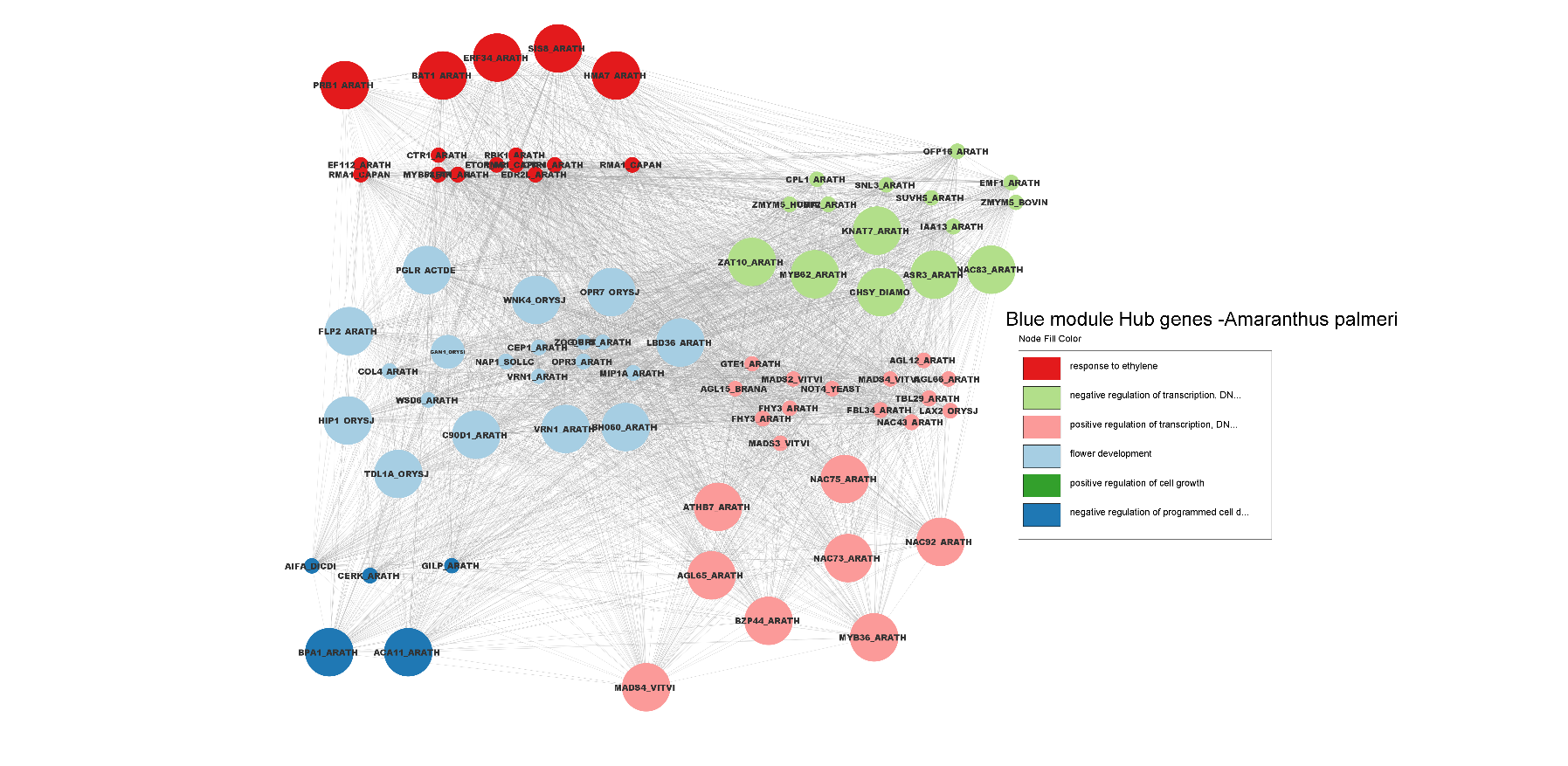


Supplemental Figure 7. Co-expression network from blue module key hubs from Amaranthus palmeri. Node size represents gene trait significance.


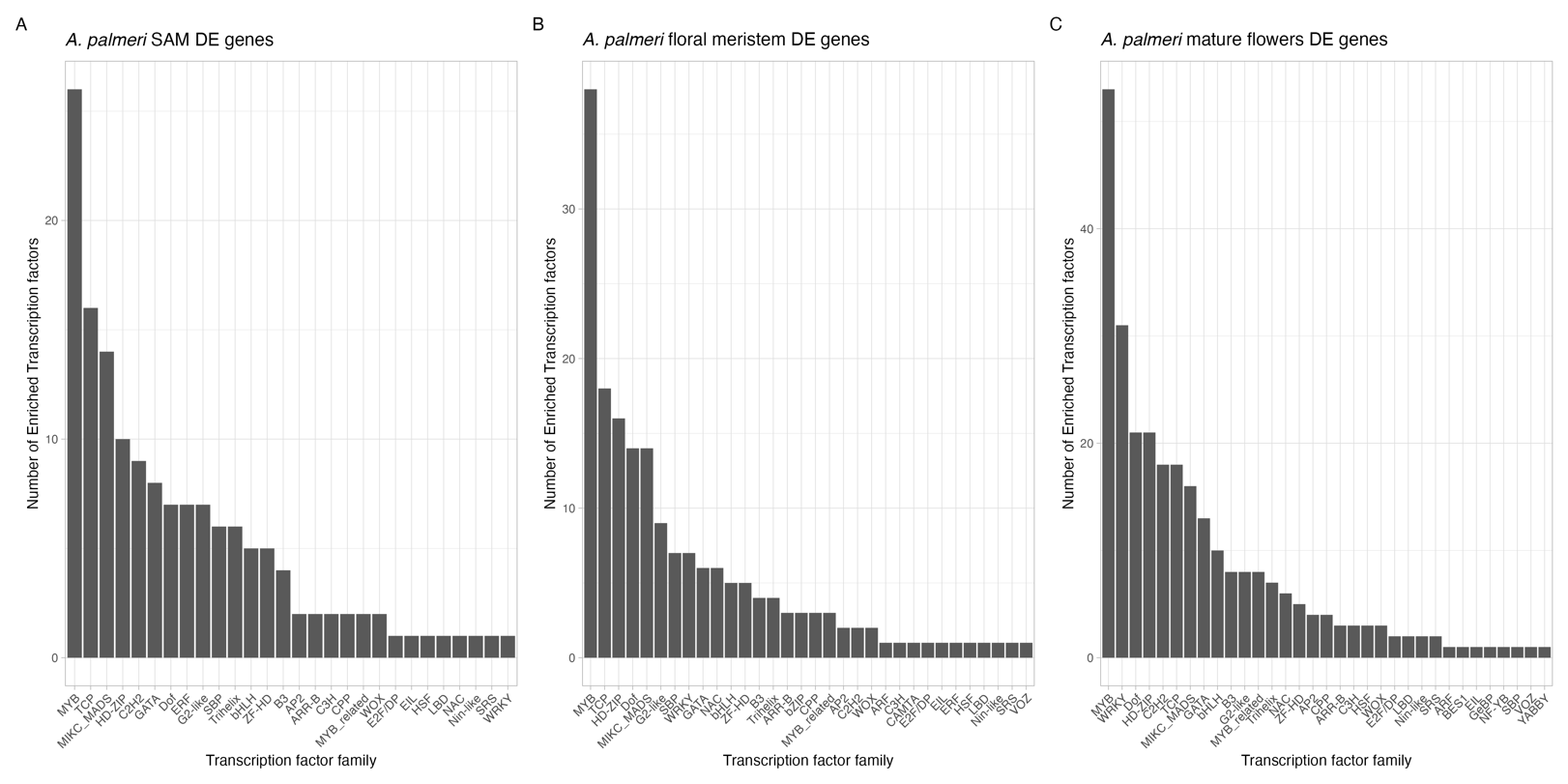


Supplemental figure 8. Promoter analysis results from A. palmeri. Analysis conducted using genes that were differentially expressed within tissues and across genders. Promoter regions extracted from reference genome were used for the analysis. Results summary of total of transcription factors per family showing motif enrichment on the extracted promoter region.

Supplemental figure 9. Principal component analysis for Amaranthus tuberculatus transcriptome data.

Supplemental figure 10. Principal component (PC) gene loading in Amaranthus tuberculatus for the first four primary PCs showing genes driving dispersion.


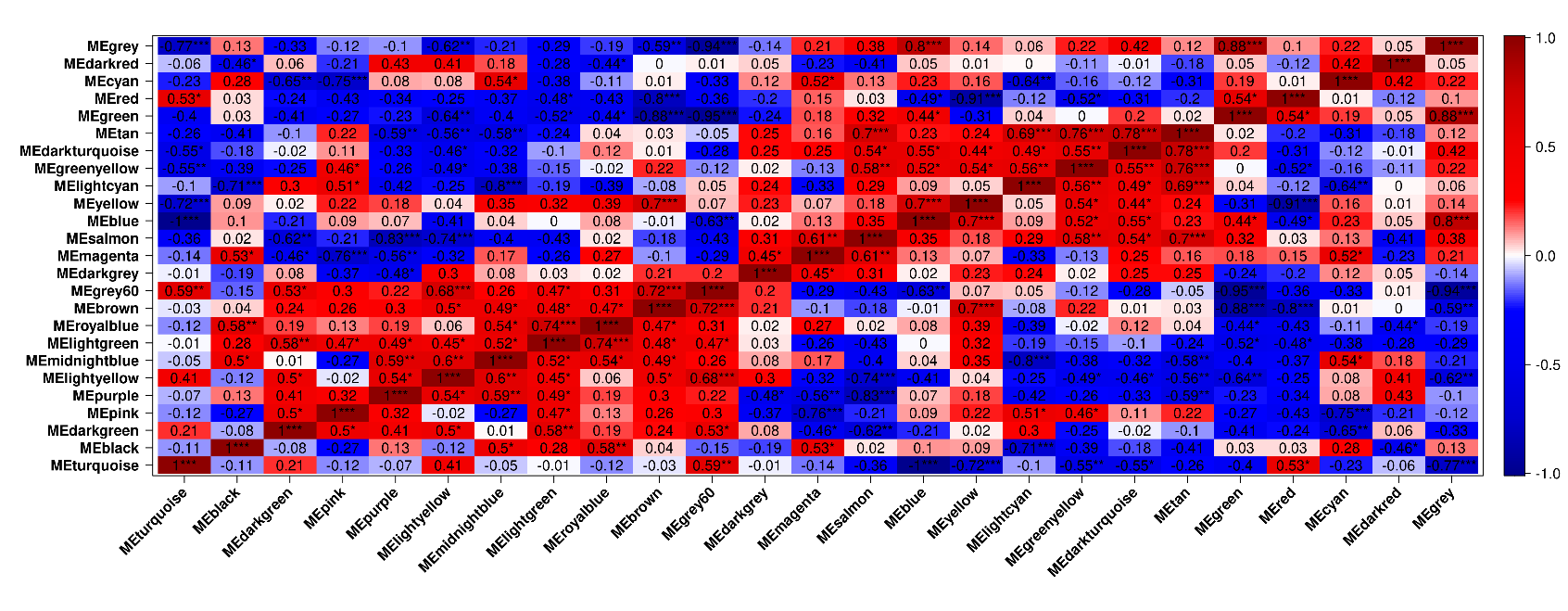


Supplemental Figure 11. Module correlation for Amaranthus tuberculatus WGCNA


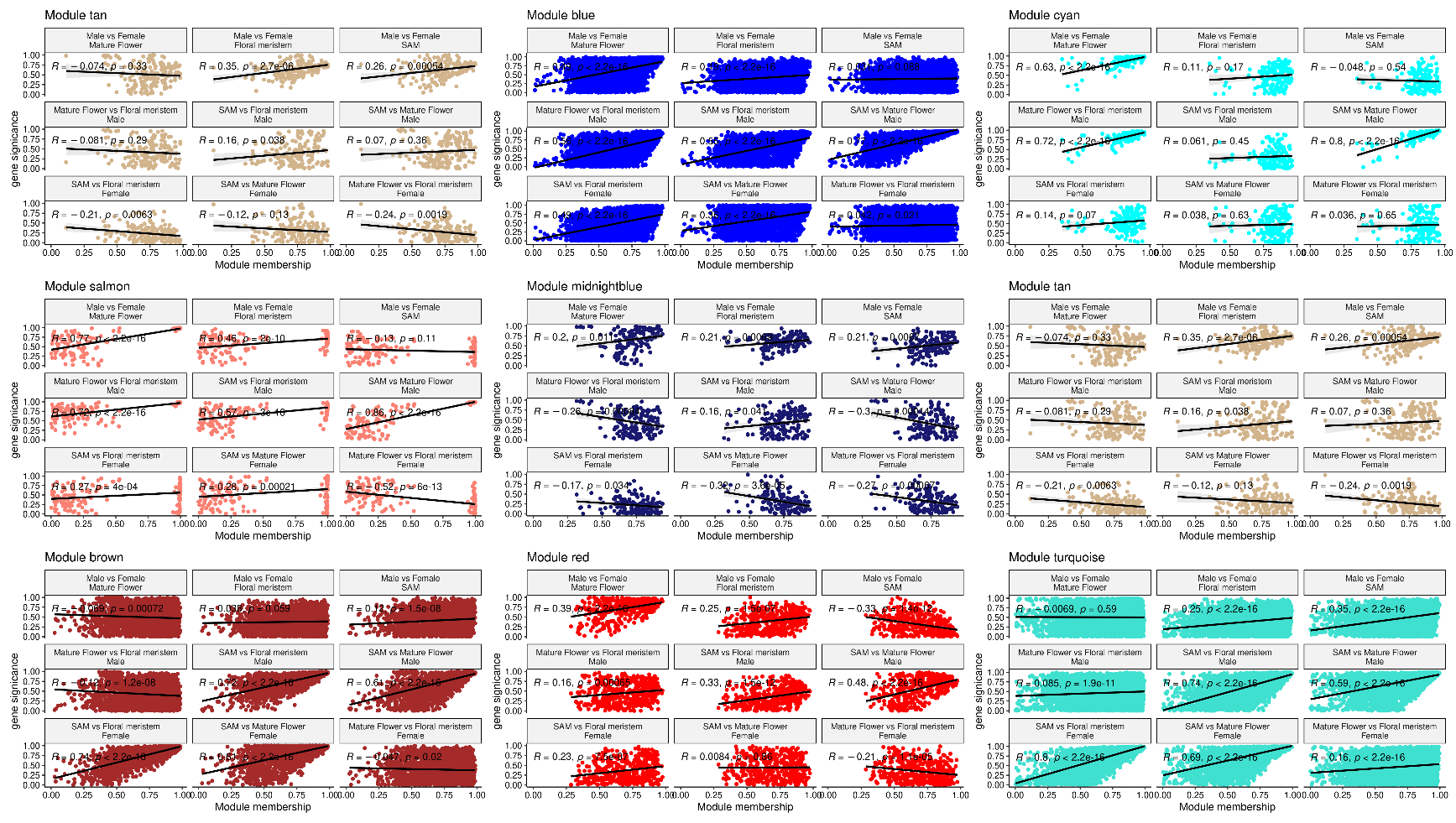


Supplemental Figure 12. WGCNA palmer intra-modular analysis to identify hub genes within main modules identified in Amaranthus palmeri analysis.


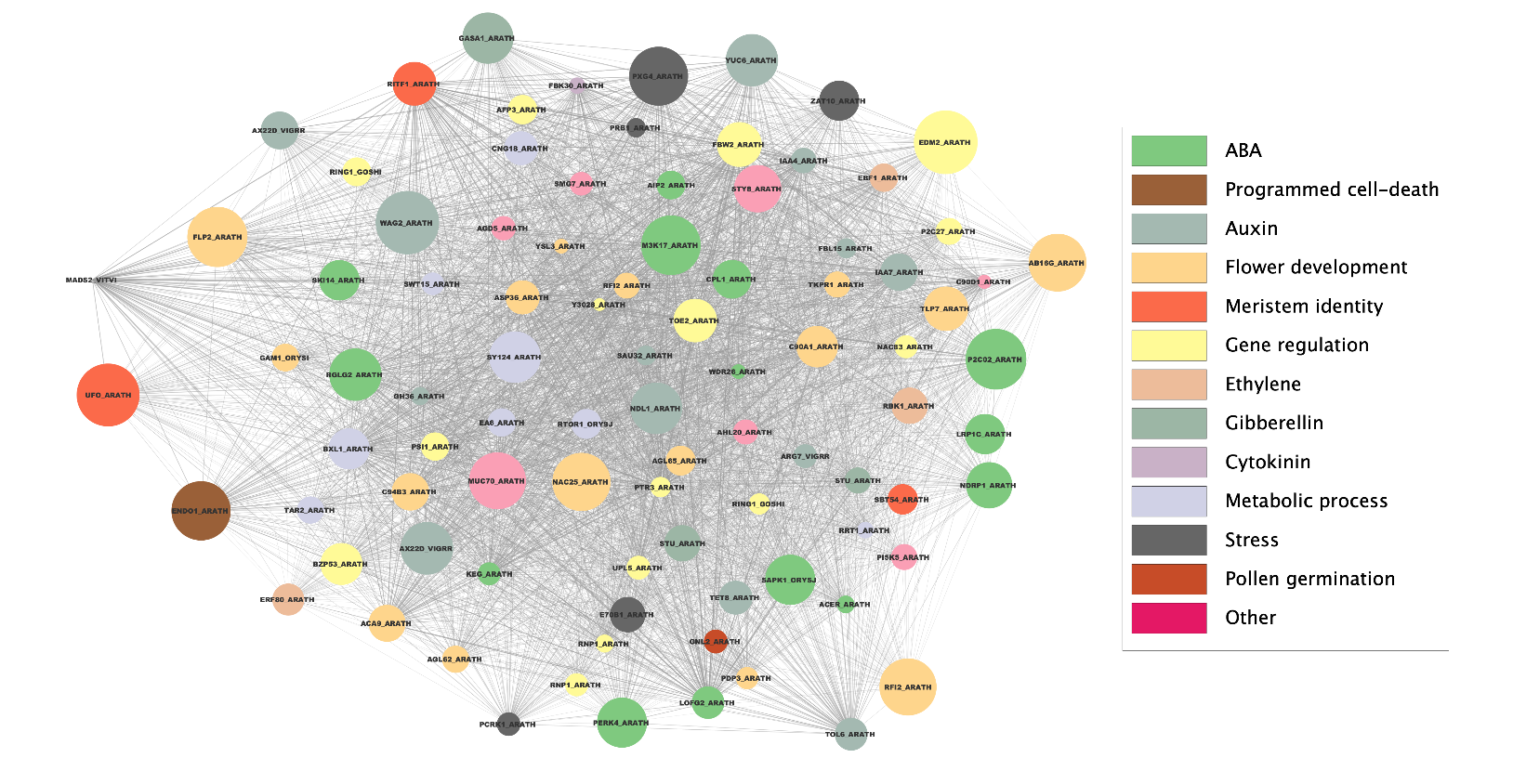


Supplemental Figure 13. WGCNA generated blue module for Amaranthus tuberculatus. Network indicates most co-expressed genes involved in putative flower development functions.


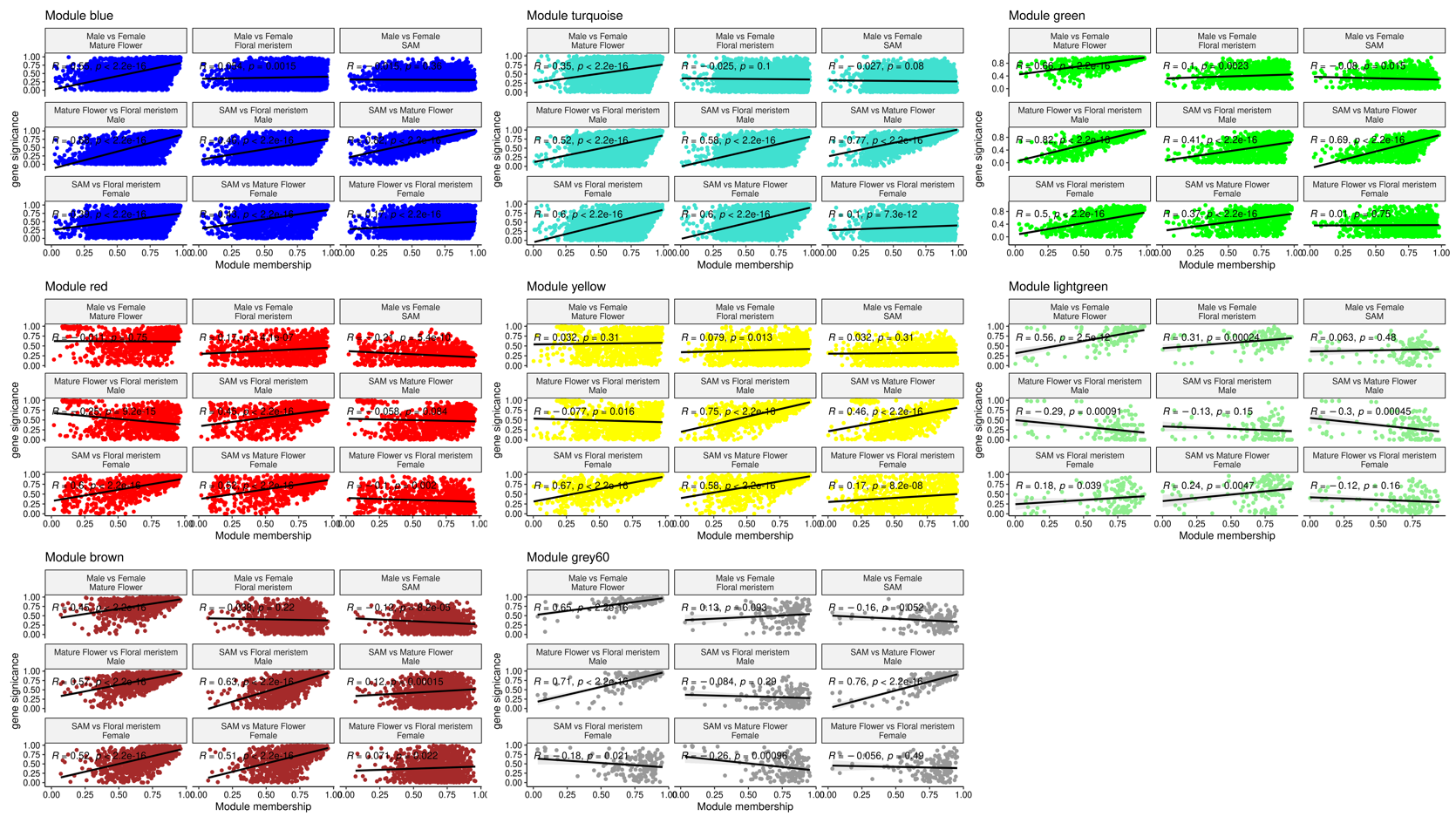


Supplemental Figure 14. WGCNA palmer intra-modular analysis to identify hub genes within main modules identified in Amaranthus tuberculatus analysis.


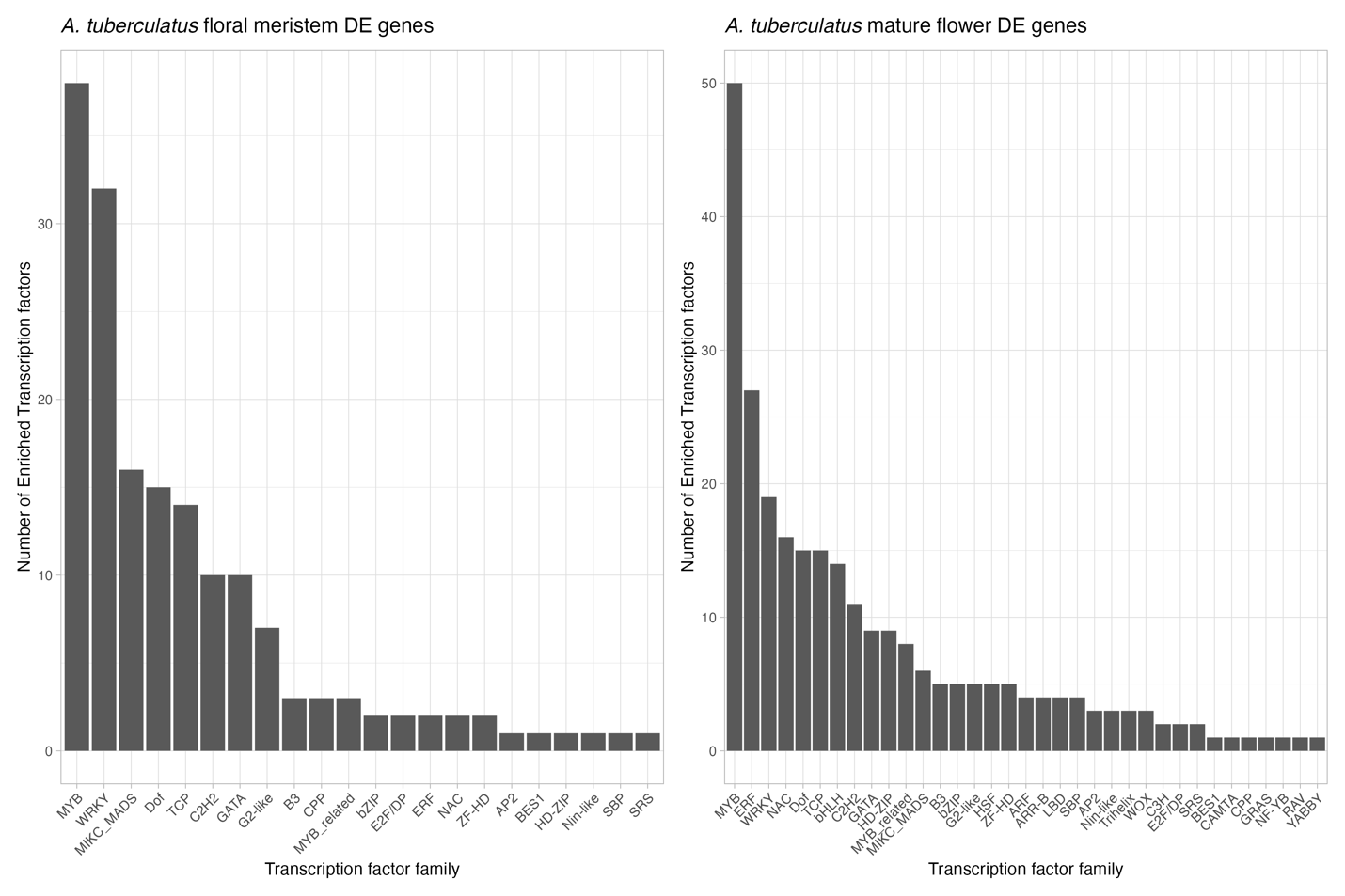


Supplemental figure 15. Promoter analysis results from A. tuberculatus. Analysis was conducted using differentially expressed genes within tissues and across genders. Promoter regions extracted from the reference genome were used for the analysis. Results summary of total of transcription factors per family showing motif enrichment on the extracted promoter region.

Supplemental Figure 16.qPCR results for RNA-seq validation.
